# Minimal mutational requirements for conversion of a telomere resolvase into a Cre-like site-specific recombinase

**DOI:** 10.1371/journal.pone.0350834

**Published:** 2026-05-29

**Authors:** Shu Hui Huang, Kerri Kobryn

**Affiliations:** Department of Biochemistry, Microbiology & Immunology, College of Medicine, University of Saskatchewan, Saskatoon, Canada; NMIMS Deemed to be University - Mumbai Campus: NMIMS, INDIA

## Abstract

Hairpin telomere resolvases comprise a family of enzymes that produce the hairpin (hp) telomeres of bacteria and their phages that possess linear chromosomes and plasmids terminated by covalently closed hp telomeres. The hp telomeres overcome the dual issues of end replication and protection faced by all organisms with linear genomes. The hp telomeres are produced from replicated intermediates in which the hp telomeres have been converted into replicated telomere (*rTel*) junctions possessing inverted repeat symmetry. The telomere resolvases process the *rTel* junctions in a reaction with mechanistic similarities to that promoted by type IB topoisomerases and tyrosine recombinases. The telomere resolvase of both *Borrelia burgdorferi* (ResT) and *Agrobacterium tumefaciens* (TelA) have been shown to be able to promote, under certain conditions, a Cre-like recombination between *rTel* junctions to produce the Holliday junction (HJ) intermediate typical of recombination reactions promoted by tyrosine recombinases. For TelA mutation of the enzyme was required to unmask this normally cryptic activity. A complex combination of autoinhibition domain deletion and point mutation of TelA had even been shown to completely switch the activity of TelA from a telomere resolvase to a recombinase. We report here that mutation of a pair of aspartic acid residues in TelA is sufficient to accomplish this switch in activity.

## Introduction

**Fig 1:**
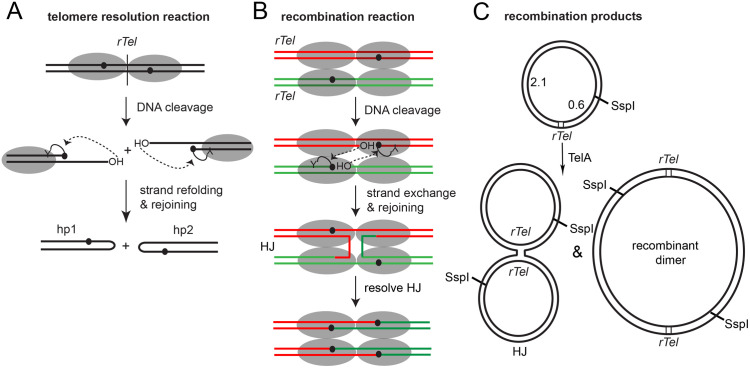
Telomere resolution and Cre-like recombination promoted by TelA. **A)** Telomere resolution involves dimerization of TelA on a replicated telomere (*rTel*) junction followed by a pair of transesterifications that result in DNA cleavage and strand rejoining (after refolding the cleaved strands) to form the hairpin (hp) telomere products. The reaction is promoted by the active site nucleophilic tyrosine (shown with a Y) using a mechanism similar to that used by type IB topoisomerases and tyrosine recombinases. **B)** Cre-like site-specific recombination involves dimerization of TelA on a pair of *rTel*s followed by their synapsis. DNA cleavage of one scissile phosphate in each *rTel* followed by strand exchange leads to the formation of a Holliday junction (HJ) that is normally transiently present intermediate of recombination promoted by tyrosine recombinases. The HJ is isomerized and undergoes a further round of DNA cleavage and strand transfer events using the second pair of scissile phosphates to produce a full, directly repeated, recombinant dimer. TelA tends to accumulate the HJ as its predominant product. **C)** Shown are graphics of the *rTel*-containing plasmid used in recombination reactions and the predominant HJ intermediate and recombinant dimer produced by reaction. For simplicity only the HJ and recombinant dimer versions of Cre-like recombination from an anti-parallel synapse are shown.

Bacteria of the genera *Borrelia* and *Agrobacterium* harbour linear replicons terminated by covalently closed hairpin (hp) telomeres. The hp telomeres help overcome the end-replication problem for linear DNA’s ([1,2] and references therein). There is also evidence that they overcome the end-protection problem [3]. While hp telomeres allow DNA replication to be completed they give rise to replicated intermediates that must be processed by a DNA cleavage and rejoining reaction, referred to as telomere resolution, in order to allow the replicated DNA to be segregated as unit-length linear replicons to the daughter cells (**Fig 1A**).

A specialized family of enzymes, the telomere resolvases, has been characterized that promote telomere resolution. The telomere resolvases share a catalytic domain and reaction mechanism for the DNA cleavage/rejoining reactions with the type IB topoisomerases and tyrosine recombinases [2,4–6]. Telomere resolution involves DNA cleavage by the active site nucleophilic tyrosine giving rise to a transient linkage of the telomere resolvase to the cleaved strands of the replicated telomere (*rTel*) junctions via a 3’-phosphotyrosine linkage ([7,8]; **Fig 1A**). The cleaved strands fold into a hairpin conformation and the 5’-OH’s are activated to attack the 3’-phosphotyrosine linkages, reforming phosphodiester bonds in the DNA, covalently sealing the cleaved *rTel* into a pair of hp telomeres. Repetition of this process at both *rTel*s in the replicated intermediate releases a pair of linear replicons terminated the hp telomeres (S1 Fig).

While type IB topoisomerases, telomere resolvases and tyrosine recombinases employ a structurally homologous catalytic domain and an essentially identical mechanism of DNA strand cleavage and rejoining, the nature of their products are very different. The type IB topoisomerases cleave and rejoin a single DNA strand while removing supercoils; consequently they act as monomers. Telomere resolvases must cleave and rejoin two strands after refolding the cleaved strands into a hairpin conformation; thus they do so as dimers assembled on an *rTel*. Tyrosine recombinases cleave and rejoin four strands in a recombinant configuration by passaging through a Holliday junction (HJ) intermediate; therefore they act as tetramers after dimerizing on a pair of specific reaction sites [2].

The underlying relationship of telomere resolution with topoisomerase (IB) and tyrosine recombinase reactions was hinted at early by sequence alignment and then revealed experimentally by conditions in which the *Borrelia burgdorferi* telomere resolvase, ResT, was discovered to promote topoisomerase relaxation of substrate plasmids or to promote site-specific recombination between *rTel* junctions (**Fig 1B & C**; [5,9]). For ResT DNA supercoiling plays an interesting role in determining the favoured reaction. Negative supercoiling suppresses telomere resolution and favours recombination [5,9]. Conversely, positive DNA supercoiling promotes telomere resolution and suppresses recombination [9,10]. Negative DNA supercoiling is also known to suppress telomere resolution promoted by TelA [6,11,12]. The wild type version of the agrobacterial telomere resolvase, TelA, has been shown to be autoinhibited to the extent that site-specific recombination between *rTel*s was undetectable [13]. Autoinhibitory interactions in TelA provided by the dispensable N-terminal domain and part of the C-terminal helix confers divalent metal-ion responsiveness on the telomere resolution reaction and prevents the competing reactions of hp telomere fusion and recombination. Disruption of these autoinhibitory interactions by deletion and mutation yielded mutants with altered metal-ion responsiveness, hyperactivity for the telomere resolution reaction and the ability to promote a small amount of recombination that was detectable by a sensitive assay [11,13].

Subsequently, a catalytic domain point mutant (D398A) of TelA was discovered to lower telomere resolution proficiency but to also massively stimulate recombination between *rTel*s to the point that it became easily detectable by multimerization of *rTel*-containing plasmids (S2 Fig in S1 File and [12]). Unexpectedly, when mutations that hyperactivated TelA for telomere resolution (typically domain deletions) were combined with the D398A point mutation a complete switch in enzyme activity was observed. What had been hyperactive telomere resolvases had become hyperactivated recombinases that could no longer promote telomere resolution [12]. These ‘switch’ mutants incorporated a complex constellation of deletions and other activating point mutations combined with the D398A mutation. Therefore, it was unclear what the minimal requirements were to produce this switched phenotype. Moreover, the resultant recombinases promoted an aberrant recombination that was unidirectional, tending to produce increasingly larger plasmid multimers. Moreover, the recombination was ‘blind’ to artificial directionality cues introduced into the *rTel*s (by asymmetrizing them between the scissile phosphates) producing HJ intermediates that when resolved by T7 endonuclease I produced products indicative of integrations to produce both inverted and direct repeat multimers [12].

In the present study we have endeavored to determine the minimal requirements for effecting a switch from a telomere resolvase that cannot promote recombination to a recombinase that cannot promote telomere resolution. We found that the minimal requirements to produce a switched recombinase phenotype is to pair the D202R mutation with the D328A mutation. The resulting D202R/D398A mutant readily combines negatively supercoiled *rTel*-containing plasmids into progressively larger multimers while showing almost no telomere resolution activity under conditions that normally promote resolution.

## Materials and methods

### DNAs

The synthetic oligonucleotides used to assemble substrates or produce site-directed mutants were purchased from Integrated DNA Technologies (IDT) and are listed in S1 Table in S1 File Telomere resolution assays used pEKK494 as a substrate. The construction of pEKK494 is reported elsewhere [13,14]. The plasmids with the asymmetric *rTel* sequences (CCATGA) and (CATTGA) between the scissile phosphates are designated as pEKK495 and pEKK592, respectively. The construction of pEKK495 was reported with that of pEKK494. pEKK592 was constructed by annealing OKBA69/70 oligos followed by BamHI-HindIII directional cloning into pUC19 as described for the construction of pEKK494 (TCATGA) and pEKK495 (CCATGA).

### Proteins

The TelA mutants reported here were generated by site-directed mutagenesis using the primers listed in S1 Table in S1 File. The induction and expression conditions are listed in S2 Table in S1 File. All TelA purifications proceeded as reported elsewhere [13,15]. BamHI, HindIII, ScaI, SspI and T7 endonuclease I were sourced from New England Biolabs.

### Plasmid-based telomere resolution and recombination assays

Telomere resolution and recombination assays were incubated at 30^o^C. The reaction buffer contained 25 mM HEPES (pH 7.6), 1 mM DTT, 100 µg/mL BSA, 50 mM potassium glutamate, 2 µg/mL of the noted plasmid substrate DNA and 50 nM TelA (unless otherwise indicated in the figures and their legends) For timecourse experiments 18 µL aliquots were withdrawn from 100 µL master reactions and the reaction was stopped by addition of 5X SDS-containing load dye to a 1X concentration. 1X load dye contains 0.2% SDS, 20 mM EDTA, 3.2% glycerol, and 0.024% bromophenol blue. The samples were loaded to 0.8% agarose 1X TAE gels that were electophoresed at 3V/cm for 3 hours. The results were visualized by staining the gels with 0.5 µg/mL ethidium bromide followed by destaining in distilled water (30 min for each step). Gel images were captured using a BioRad GelDoc system. When recombination reactions were analyzed using restriction digest and treatment with T7 endonuclease I the reactions were heat killed at 65^o^C for 20 min. The MgCl_2_ concentration was adjusted to 10 mM prior to addition of 2.5 units of SspI (37^o^C, 1 h incubation) and where indicated subsequent addition of 3 units of T7 endonuclease I (30^o^C, 2 min incubation). Recombination analysis reactions were terminated by addition of SDS-load dye to a 1X concentration. Where indicated in the legend samples supplemented with pronase were treated with 100 µg/mL pronase (37^o^C, 5 min) prior to gel loading. The samples were loaded to 0.8% agarose 1X TAE gels that were electophoresed at 3V/cm for 3 hours.

### Recombination assays between plasmid and fluorescein-labeled small synthetic substrates

The ability of the TelA (D202RD398A) mutant to promote recombination between an *rTel* on a plasmid and a small synthetic *rTel* was assayed using negatively supercoiled pEKK494 or pEKK592 and 5’-fluorescein end-labeled *rTels* of the same sequence (and heterologous flanks). For reactions with pEKK494 76 nM of TelA (D202R) were reacted with 10 µg/mL of pEKK494 and 40 nM oligonucleotide *rTel* (OKBA75F/OGCB951F) in our standard telomere resolution reaction buffer with 2 mM MgCl_2_ (see above). For reactions with pEKK592 304 nM of TelA (D202R) was reacted with 10 µg/mL of pEKK592 and 40 nM oligonucleotide *rTel* (OKBA69F/OKBA70F). Control incubations omitted TelA. Reactions were incubated at 30^o^C for 2 h followed by heat treatment (75^o^C, 20 min) of an aliquot of the reaction with TelA, substrate plasmid and *rTel* all present to mimic branch migration of the resulting HJs into linear recombinant product. In order to load the samples to a gel the reactions were terminated by addition of 5X SDS loading dye to a 1X final concentration prior to gel loading. For reaction with pEKK592 pronase treatment was also required prior to gel loading (100 µg/mL pronase incubated at 37^o^C for 10 min). Reactions were loaded to 0.8% (w/v) agarose 1X TAE gels and electrophoresed at 3.25V/cm for 3 h. The bottom of the gels with the free unreacted *rTel*s were cut off and then gels were re-imaged for fluorescein labeled material. Subsequently, gels were stained with 0.5 µg/mL ethidium bromide and visualized on a Biorad GelDoc system using the ethidium bromide program to visualize the plasmid substrate and molecular weight markers.

For reactions that were loaded to alkaline agarose gels the reactions were heat killed at 65^o^C for 20 min prior to adjusting the MgCl_2_ concentration to 10 mM followed by addition of 20 units of ScaI with incubation at 37^o^C for 60 min. For reactions conducted with pEKK592 and its synthethic *rTel* counterpart pronase was added to 100 µg/mL followed by incubation at 37^o^C for 10 min. 5X alkaline load dye was added to a 1X concentration and the samples were loaded to a 0.8% agarose 1X alkaline gel and electrophoresed at 1V/cm for 19 h. 1X alkaline load dye contains 50 mM NaOH, 1 mM EDTA, 0.04% xylene cyanol and 3% Ficoll. The entire gel was imaged in the fluorescein channel after cropping out the xylene cyanol and unreacted synthetic *rTel* signals which fluoresced near the top and bottom of the gels, respectively. The 1 kb ladder lane was cropped, neutralized and stained in 0.5 µg/mL ethidium bromide, placed back next to the remainder of the gel and then imaged in the ethidium bromide channel using a Biorad GelDoc system. The GelDoc system generated a composite image for presentation.

## Results

### Combining two point mutations is sufficient to switch TelA from a telomere resolvase to a Cre-like site-specific recombinase

Previous studies with recombination proficient TelA mutants showed that by combining autoinhibition domain deletions, activating point mutations and the D398A mutation in the catalytic domain led to the activation of recombination accompanied by the complete suppression of the telomere resolution activity of the enzyme; effectively switching the activity of TelA. The ‘switch’ mutants incorporated a combination of activating domain deletions and a pair of point mutations (D202R and E337K) added to the D398A mutation that permits recombination. We wondered if the domain deletions were really necessary for the switch phenotype. To examine this we looked at the properties of the D202R, E337K, D202R/E337K, D202R/D398A, E337K/D398A and D202R/E337K/D398A mutants. The rationale for the use of an alanine residue at D398 and charge reversal mutations at D202 and E337 derive from their being originally made in a general alanine scanning screen of conserved catalytic domain residues of unknown function for D398 [16] and an interrogation of hypothesized salt bridge interactions supposed to establish autoinhibitory interfaces in TelA, respectively [11]. The analysis began with an examination of the basic folding of the proteins via the proxy of retention of the single-stranded DNA annealing activity known to be possessed by both ResT and TelA (S3 Fig in S1 File and refs [11,13,15,17]). No significant defect in annealing was noted for any of the mutant proteins suggesting they were not misfolded. The analysis of these mutants continued with examination of the basic parameter of the ability to bind telomeres (S4 Fig in S1 File). Because the D202R and E337K mutants are hyperactive for telomere resolution even at 0^o^C, we performed electrophoretic mobility shift assays (EMSA) of all the mutants with 5’-fluorescein labeled hp telomeres rather with *rTel* substrate since the D202R and E337K mutants would resolve the *rTel*s complicating the interpretation of the results (S4 Fig in S1 File and ref. [11]). Both the telomere resolution activating D202R and E337K mutants had a slightly higher affinity for hp telomere than wild type TelA while the recombination activating D398A mutant showed lower affinity for the hp telomere than wild type TelA. The D202R/D398A and E337K/D398A mutants showed modestly higher hp telomere affinity relative to D398A. Also evident from the EMSA’s is that the bandshifts came as multiple bandshifted species implying that beyond TelA binding to a hp telomere there was differing abilities of the tested mutants to multimerize (S4 Fig in S1 File).

**Fig 2:**
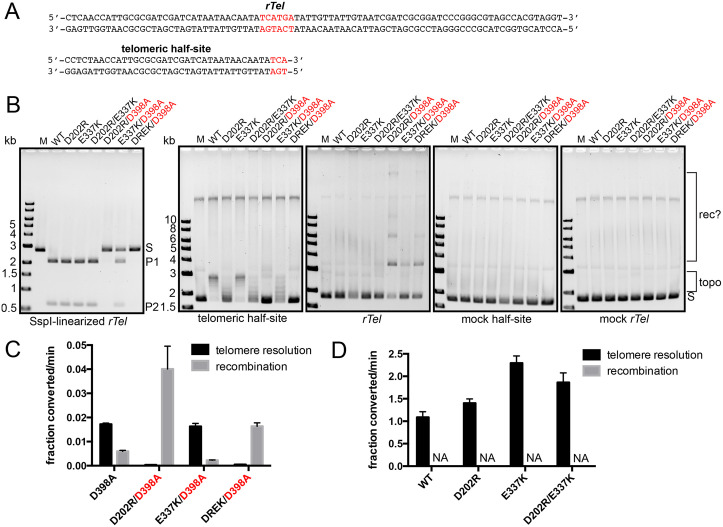
Determination of the minimal requirements for TelA hyperactivation and conversion into a site-specific recombinase. **A)** The sequences of the replicated telomere junction (*rTel*) and the telomeric half-site are shown. The half-sites are made up of one copy of the symmetry element that makes up the inverted repeat found in the *rTel*. Mock versions systematically changed A’s to T’s, G’s to C’s (and *vice versa*) to maintain the sequence composition and secondary structure while changing the sequence of the substrates. These sequences were directionally cloned into pUC19 as detailed in the Materials and Methods section. **B)** 0.8% agarose 1X TAE gel panels of incubations with 50 nM of the noted TelA mutants *vs.* wild type TelA using 2 µg/mL of pUC19 variants with telomeric half-site and *rTel* inserts. Also shown are control panels with mock half-site and mock *rTel* versions of the plasmids. All incubations were at 30^o^C for 30 min in a buffer containing 2 mM MgCl_2_. S denotes substrate; P1 & P2 denote the products of telomere resolution; topo denotes the ladder of topoisomers; rec? denotes plasmid multimers likely composed of HJ’s and full recombinants. For labeling the mutants shown above the gel-loading key the triple mutant shortens the D202R/E337K portion of the triple mutant to DREK. **C)** Summary graph of the initial rates of telomere resolution *vs.* recombination for the recombination proficient mutants tested with 50 nM TelA. **D)** Summary graph of the initial rates of telomere resolution *vs.* recombination of the mutants lacking the D398A mutation needed to activate recombination; the mutants were tested using 50 nM TelA. NA indicates that no activity was detectable at the longest timepoint tested (2 h). Shown are the mean and standard deviation of three independent trials of each experiment.

Having examined the TelA mutants for these basic parameters we used them in reactions with plasmid substrates for telomere resolution, recombination and topoisomerase relaxation as well as control versions with mock telomeric sequences in place of the genuine telomere sequences. The mutants (and wild type TelA) with only the activating mutations D202R and E337K were active in telomere resolution assayed with the SspI-linearized *rTel*-containing plasmid but were inactive in the recombination assay with supercoiled *rTel* plasmid (**Fig 2**). With a plasmid harbouring only half of the inverted repeat *rTel* wild type TelA and the E337K mutant had almost fully relaxed this plasmid while the D202R mutant showed little topoisomerase activity with the conditions tested. When the D398A mutation that activates recombination was added to the activating mutant backgrounds two variants that were switch mutants were obtained; being active for recombination but inactive for telomere resolution (D202R/D398A and D202R/E337K/D398A; **Fig 2B**). The pairing of the E337K mutation with the D398A mutation produced a double mutant with reduced telomere resolution proficiency that was inactive for recombination. Interestingly, the two switch mutants showed no topoisomerase activity on the telomeric half-site plasmid. In all cases there was no activity noted on the negative control mock telomere and mock half-site plasmid confirming the sequence specificity of all observed positive results (**Fig 2B**).

A closer examination of the reaction rates of the recombination-proficient mutants revealed that the two switch mutants (D202R/D398A and D202R/E337K/D398A) were activated for recombination compared to the D398A mutation alone and were almost completely inactive for telomere resolution. The D202R/E337K/D398A mutant is a slower recombinase than the simpler D202R/D398A mutant indicating that combining the D202R and D398A mutations is sufficient, indeed optimal, to produce the switch phenotype. Combining the E337K mutation with the D398A mutation did not activate (or suppress) telomere resolution and produced a slower recombinase than the D202R/D398A and D398A mutants (**Fig 2C**). Finally, wild type TelA and the mutants that have activating point mutations were assayed using timecourses and the activation specific to telomere resolution was confirmed. The E337K mutation that increases the affinity for substrate was optimally activating and was not additive with the activating D202R mutation that helps reduce the divalent metal ion dependence of telomere resolution ([11]; and **Fig 2C**).

**Fig 3:**
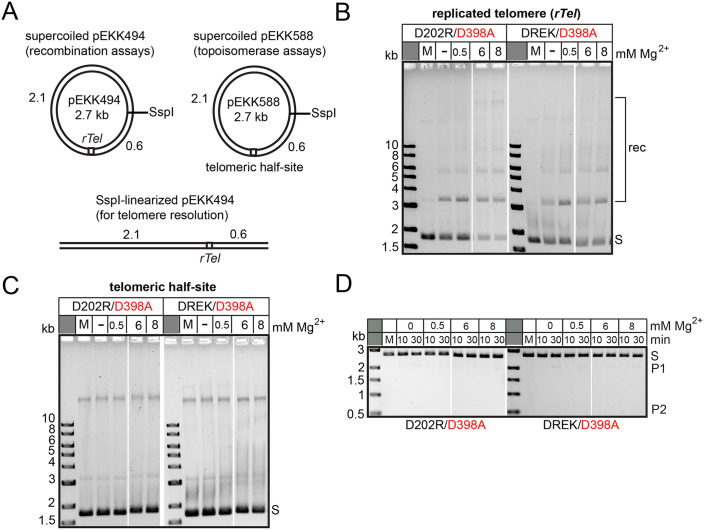
Analyzing the hyperactive recombinases for their responsiveness to divalent metal-ion concentration. **A)** Schematics of the structure of the plasmid substrates used for the recombination, topoisomerase and telomere resolution assays, respectively. The position of the single-hit restriction site for SspI relative to the position of the cloned *rTel* and half-site inserts is shown. The inserts divide the plasmid substrates into 2.1 kb and 0.6 kb domains relative to the position of the SspI site. The sequence of the *rTel* and telomeric half-site inserts present in the plasmids is detailed in Fig 2A. **B)** 0.8% agarose 1X TAE gel panels of divalent metal ion titrations of the D202R/D398A and D202R/E337K/D398A mutants reacted with negatively supercoiled *rTel* plasmid substrate (pEKK494) incubated at 30^o^C for 30 min in a buffer containing the indicated concentrations of MgCl_2_. The 0, or no Mg^2+^ condition, contains 1 mM EDTA instead. **C)** 0.8% agarose 1X TAE gel panel of divalent metal ion titrations of the D202R/D398A and D202R/E337K/D398A mutants reacted with negatively supercoiled telomeric half-site plasmid substrate (pEKK588) incubated at 30^o^C for 30 min in a buffer containing the indicated concentrations of MgCl_2_. The 0, or no Mg^2+^ condition contains 1 mM EDTA instead. S denotes substrate; rec denotes multimers of recombined plasmids. **D)** 0.8% agarose 1X TAE gel panels of divalent metal ion titrations of the D202R/D398A and D202R/E337K/D398A mutants reacted in telomere resolution reactions with SspI-linearized wildtype plasmid substrate (pEKK494) at 30^o^C for 10 min and 30 min timepoints in a buffer containing the indicated concentrations of MgCl_2_. S denotes substrate; P1 & P2 denote the migration position of the expected products of telomere resolution.

The two ‘switch mutants’ D202R/D398A and D202R/E337K/D398A were subjected to a more thorough characterization of potential recombination, topoisomerase and telomere resolution activities over a range of additional MgCl_2_ concentrations. In contrast to the behaviour of the D398A single mutant that requires MgCl_2_ to display recombination activity the two switch mutants promote divalent metal ion independent recombination (**Fig 3**; [12]). However, adding MgCl_2_ did result in conversion of more substrate plasmid into recombination products (**Fig 3**). The switch mutants remained inactive in topoisomerase and telomere resolution assays even at the highest concentration (8 mM) of MgCl_2_ tested (**Fig 3**). The remaining mutants that did not promote recombination were subjected to the same analysis over a range of additional MgCl_2_ concentrations (S5-S6 Figs in S1 File). They all remained inactive for recombination in the expanded range of MgCl_2_ concentrations. The D202R and D202R/E337K mutants that showed very limited topoisomerase activity at 2 mM of MgCl_2_ supported almost full relaxation of the telomeric half-site plasmid at 6 and 8 mM of MgCl_2_ (**Fig 2A**). For telomere resolution wild type TelA and the E337K mutant displayed the greatest response to added MgCl_2_ while the D202R/E337K and E337K/D398A mutants showed little response to increasing MgCl_2_ concentrations (S5-S6 Figs in S1 File).

### Recombination by the switch mutants features accumulation of HJ’s

**Fig 4:**
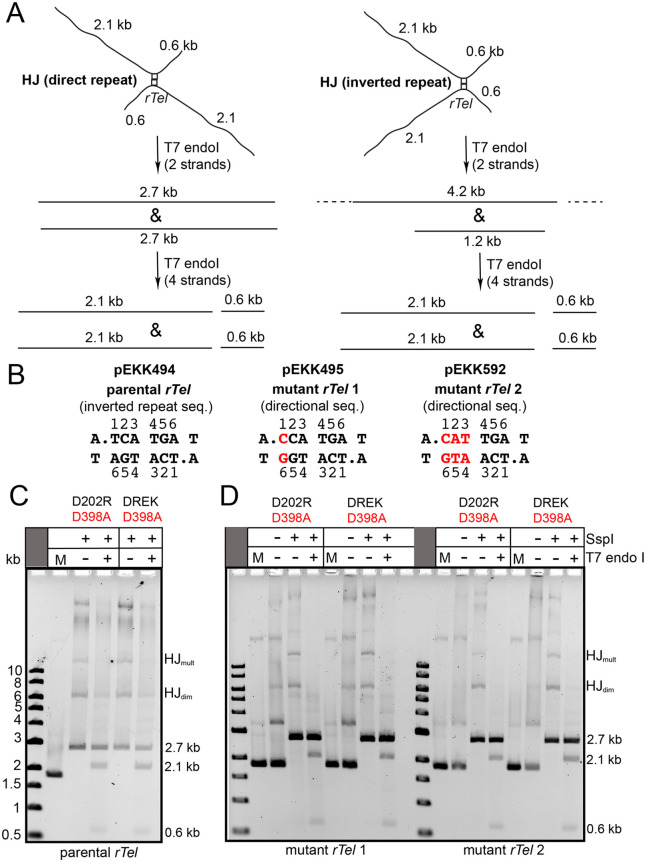
Analysis of the HJ produced by the recombinase mutants with plasmid reactions: determining if asymmetrizing the *rTel* sequence allows for reaction directionality control. **A)** Representation of the expected structure of the HJs resulting from alternate orientations of synapsis and strand exchange. Also shown are the expected results from incubation with a HJ resolvase (T7 endo I) in the cases where two strands or four strands of the HJ have been cleaved. A version of the graphic originally appeared in our previous paper, held under a creative commons license (ref [12]). **B)** Shown is the sequence between the scissile phosphates for the parental *rTel* sequence with complete inverted repeat symmetry *vs.* two mutant *rTel*s that disrupt the inverted repeat symmetry of the parent via mutation(s) introduced between the scissile phosphates (mutant *rTel* 1; pEKK495) and (mutant *rTel* 2; pEKK592). **C)** 0.8% agarose 1X TAE gel panel of the reaction of the D202R/D398A and D202R/E337K/D398A mutants with the parental *rTel* (pEKK494) employing 30^o^C, 30 min and 60 min incubations, respectively, in a buffer with 2 mM MgCl_2_ followed by digestion of the reactions with SspI and by treatment at 37^o^C, 10 min with T7 endonuclease **I.** Gels labels are as noted in **D)**. D) 0.8% agarose 1X TAE gel panel of the reaction of the D202R/D398A and D202R/E337K/D398A mutants with mutant *rTel* 1(pEKK495) and mutant *rTel* 2 (pEKK592) employing 30^o^C, 2 h incubations in a buffer with 2 mM MgCl_2_ followed by digestion of the reactions with SspI and by treatment at 37^o^C, 6 min with T7 endonuclease **I.** M denotes a mock incubation of supercoiled substrate plasmid without TelA addition or subsequent treatment; HJ denotes Holliday junctions; the numbers to the right of the gel indicate the size of the products noted in kb. The ethidium bromide stained gel is shown as an inverted image.

Our previously characterized switch mutants were found to accumulate HJs. HJs are normally a difficult to visualize transient intermediate in tyrosine recombinase reactions [12]. Furthermore, we observed that the switch mutants executed strand transfer between *rTel*s from both anti-parallel (the norm) and parallel orientations leading to the production of integration to produce direct repeats and inverted repeats, respectively, when the HJ’s were resolved by the HJ resolvase T7 endonuclease I. This was the case even when the *rTel* junctions were asymmetrized by disrupting the perfect inverted repeat sequence between the scissile phosphates [12]. Such asymmetric sequences provide directionality cues in simple tyrosine recombinases like Flp or Cre where strand exchange is restricted to exchange between reaction sites arranged in an anti-parallel synapse [18–20]. We wondered if our minimally mutated switch mutants would also be blind to directionality cues or would display more standard recombinase properties. To investigate this we employed our switch mutants in recombination reactions with plasmid substrates with a parental *rTel* and two mutant *rTel*s with progressively greater deviation from the parental *rTel*’s inverted repeat symmetry. These reactions were then linearized with SspI to visualize the accumulated HJs and these were, in turn, resolved with T7 endonuclease I (Figs 4A-D). Multiple HJs were produced by the reactions of both switch mutants with all three plasmid substrates. The predominant resolution product obtained with reaction with T7 endonuclease I were the 2.1 kb and 0.6 kb fragments indicative of resolution of the HJs by cleavage of all four strands rather than just via two strands (Fig 4A & D). Consequently, little can be concluded about the symmetry of the reaction. This 4-strand resolution was a feature of the previous study and is probably caused, in part, by the unusual symmetry of the resulting HJs [12,21,22].

### The optimal switch recombinase TelA (D202RD398A) can form HJ’s by strand exchange between supercoiled substrate plasmid and a small synthetic linear substrate *rTel*

**Fig 5:**
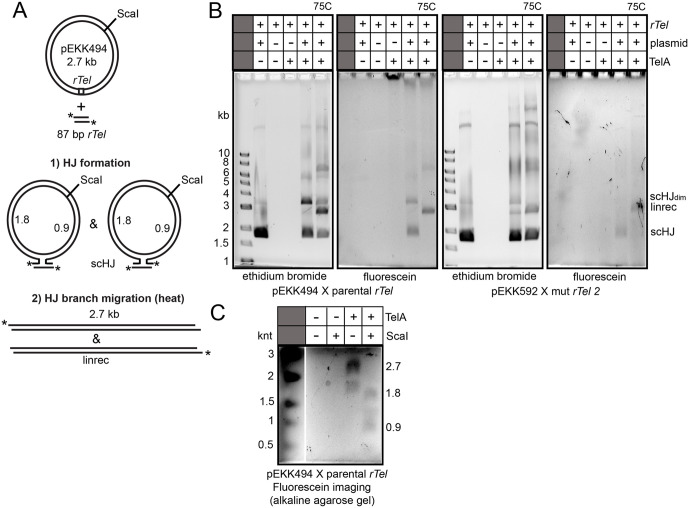
HJ formation in recombination reactions between plasmid substrates and small synthetic *rTel*s: determining if asymmetrizing the *rTel* sequence allows for reaction directionality control. **A)** Schematic of the reaction between a plasmid carrying an *rTel* junction and a 5’-fluorescein endlabeled *rTel* assembled with oligonucleotides. The asterisks (*) indicate the position of the 5’-fluorescein endlabels. 1) The two possible orientations of the resulting HJs is shown, assuming a non-directional recombination reaction using a wild type *rTel* junction with complete dyad symmetry. 2) shows the product expected from the HJs when they are disrupted by heating the resultant HJs to 75^o^C prior to gel loading. This mimics branch migration to a linear recombinant product. A version of the graphic originally appeared in our previous paper, held under a creative commons license (ref [13]). **B)** 0.8% agarose 1X TAE gel panels of reactions of the TelA (D202RD398A) mutant with pEKK494 (parental *rTel*) and pEKK592 (mutant *rTel* 2) with 5’-fluorescein endlabeled small synthetic *rTel*s of the same sequence (with heterologous flanks). The reaction conditions were optimized separately for the two substrate combinations as noted in the Materials and Methods section. scHJ; denotes a supercoiled HJ formed between the oligo *rTel* and plasmid *rTel* (also the migration position of supercoiled substrate plasmid), linrec; denotes the linear product, with a fluorescein label, of heat disruption of the scHJ, scHJ_dim_; denotes the migration position of the product of strand exchange between plasmids to make a dimer, 75C; denotes the samples that were heat treated prior to gel-loading. Beneath the gel panels is shown the identity of the recombining *rTel*s. The sequence of the *rTel*s is shown in Fig 4B. **C)** 0.8% agarose 1X alkaline gel panel of reactions of the TelA (D202RD398A) mutant with pEKK494 (parental *rTel*) with a 5’-fluorescein endlabeled small synthetic *rTel* of the same sequence (with heterologous flanks). The strand lengths (in kilonucleotides) are noted on the sides of the gel. The 1 kb ladder was cropped and ethidium bromide stained for imaging while the remainder of the gel was not stained but instead visualized using the fluorescein channel. A composite image of the ladder and experiment is presented.

The 4-stranded resolution of the resulting HJs by T7 endonuclease I precluded obtaining useful information on the orientation of synapsis for the recombining *rTel*s. We conducted preliminary trials with an alternative method of visualizing recombination that utilizes reactions between a synthetic *rTel* with 5’-fluorescent labels on both strands and negatively supercoiled substrate plasmids carrying an *rTel* of the same sequence (**Fig 5**). Since D202R/D398A was the optimal recombinase we restricted our subsequent analysis to this mutant. Reactions with D202R/D398A and the parental *rTel vs.* mutant *rTel* 2 combinations confirmed that HJ’s were produced in both cases. Reaction with the mutant *rTel* 2 that has complete disruption of the inverted repeat symmetry between the scissile phosphates required a quadrupling of the TelA concentration to achieve reaction levels comparable to the parental *rTel* (**Fig 5B**). The HJs produced migrate at the position of the supercoiled substrate plasmids indicating that DNA cleavage and strand exchange was a concerted process not allowing time for the release of plasmid supercoiling. In order to obtain an estimate of the yield of the HJs produced we heat treated an aliquot of the HJs to force the disruption of the HJs to linear products. This mimics the branch migration of the HJs to linear recombinant (**Fig 5A**). We were able to visualize HJs formed by strand exchange between both the parental and mutant *rTel* 2 pairings (**Fig 5B**). However, for the mutant *rTel* 2 pairing it was necessary to treat the terminated reactions with pronase prior to gel loading. Omission of this step resulted in the accumulation of strand exchange products that were stuck in the well. We infer that the mutant *rTel* 2 sequence promotes formation of a nicked version of the HJ that TelA is covalently attached to at the time of reaction termination. The large net positive charge of TelA was likely what prevented the products from migrating into the agarose gel until pronase treatment removed the attached TelA.

We obtained adequate levels of HJ formation with the parental *rTel* pairing to justify the attempt to analyze the HJs via restriction digest with the single-hit enzyme ScaI followed by visualization via alkaline agarose gel electrophoresis (**Fig 5C**). As expected for this symmetric substrate sequence an equivalent yield of the two possible strand lengths resulting from strand exchange to produce HJs was obtained. This indicates that synapses of the plasmid and synthetic *rTel*s in both possible orientations led to the strand exchange the produced the HJ (see schematic in **Fig 5A**). Unfortunately, we were not able to conduct a similar analysis with the HJ produced with the mutant *rTel* 2 pairing. The necessity to terminate the reaction with SDS and to pronase treat the products, in SDS, produced an irreconcilable incompatibility with the alkaline gel buffer conditions needed to visualize strand lengths in the HJ product. Therefore, we remained uncertain whether TelA (D202RD398A) was blind to the directionality cue provided by the sequence of the mutant *rTel* 2 substrate. We suspect so, since the simpler switch mutant has not eliminated the aberrant accumulation of HJs or the unidirectional nature of recombination that produces multimers increasing size in prolonged reactions.

### Characterization of the mutants that activate telomere resolution

**Fig 6:**
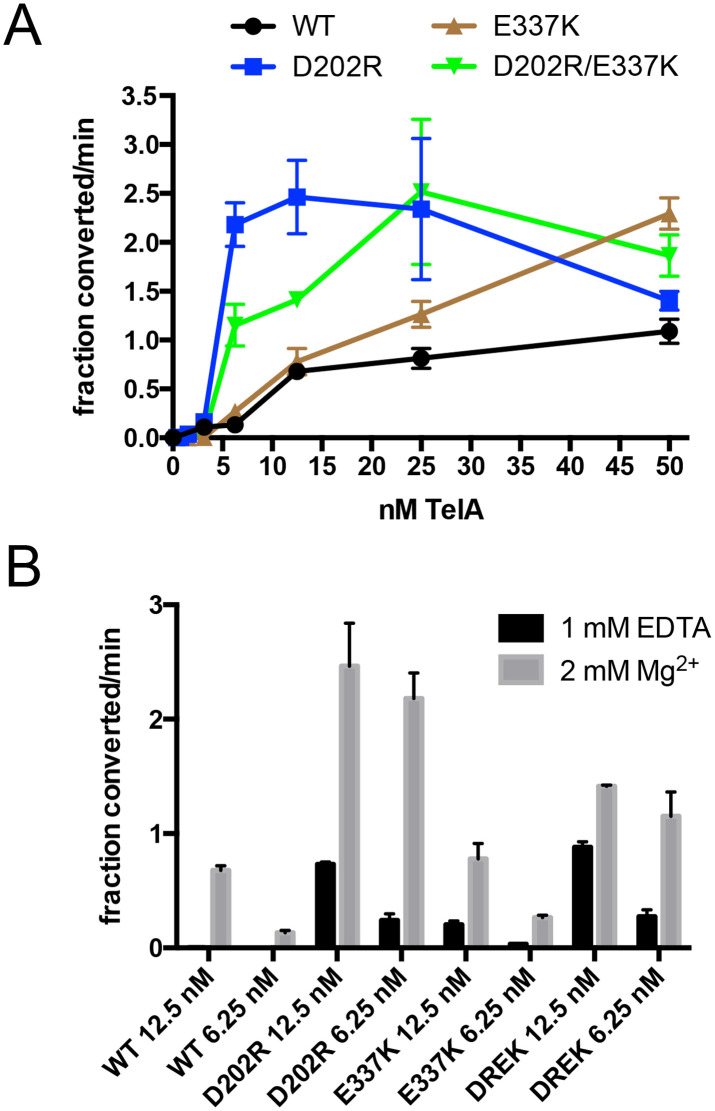
Analysis of the TelA mutants activated for telomere resolution. **A)** Summary graph of the initial rates of telomere resolution for wild type TelA (WT) and the hyperactive TelA mutants plotted against TelA concentration. The buffer conditions utilized include 2 mM MgCl_2_. Shown are the mean and standard deviation of three independent trials of each experiment. **B)** Summary graph of the initial rates of telomere resolution with wild type TelA and the hyperactive mutants plotted for reactions containing 12.5 nM and 6.25 nM TelA comparing reaction rates obtained in buffers containing 1mM EDTA *vs.* 2 mM MgCl_2_. Shown are the mean and standard deviation of three independent trials of each experiment.

The D202R and E337K mutants were developed in an attempt to rationally hyperactivate TelA by disrupting putative autoinhibitory interactions, modeled by AlphaFold2, established between several pairs of oppositely charged residues facing each other in the modeled autoinhibitory interfaces. While the specific modeled interactions were not supported by experiments that tested the existence of the specific modeled salt bridges, the charge reversal mutations were found to be useful by providing activation of the telomere resolution reaction [11]. Since these activating mutations were both present in the complicated switch mutants and a full characterization of the stimulation of the telomere resolution reaction afforded by these mutants has yet to be completed we conducted a fuller characterization of these mutants. To this end we assessed the degree of activation afforded by the D202R, E337K mutations and the double mutant by comparing the initial rate of telomere resolution promoted by these mutants with that of wild type TelA over a range of enzyme concentrations (**Fig 6A**). Wild type TelA had slow rates at lower protein concentrations (below 12.5 nM) essentially plateauing after that. The initial rates of telomere resolution promoted by E337K continues to increase linearly over the concentration range tested. The D202R mutant showed much faster initial rates at low TelA concentrations with 3.6-fold and 16.4-fold faster rates at 12.5 and 6.25 nM concentrations, respectively compared to wild type TelA. The D202R/E337K mutant showed 2.1-fold and 8.6-fold faster rates at 12.5 and 6.25 nM concentrations, respectively, compared to wild type TelA. Both the D202R and D202R/E337K mutants show a TelA concentration optima of 12.5 and 25 nM, respectively, over which the rate of telomere resolution slows (**Fig 6A**).

Mutation of the D202 residue to either alanine or arginine or otherwise interrupting autoinhibitory interactions (by, for example, deleting the N-terminal autoinhibitory domain) in TelA has been previously implicated in lowering the divalent metal ion dependence of telomere resolution [11,13]. We examined this effect with an examination of the initial rates of telomere resolution promoted by wild type TelA and the activating mutants at 12.5 and 6.25 nM TelA concentrations where the stimulatory effect of the D202R and D202R/E337K mutants was greatest in reactions with buffers containing no divalent metal ions (1 mM EDTA) *vs.* buffers with 2 mM MgCl_2_ (**Fig 6B**). In metal-free buffer telomere resolution by wild type TelA was undetectable while the D202R mutant supported rapid telomere resolution at both 12.5 and 6.25 nM TelA concentrations in metal-free conditions. Addition of MgCl_2_ was still stimulatory (3.36- and 9.1-fold stimulation relative to metal-free conditions) implying that a substantial degree of autoinhibition still exists in this mutant. The E337K mutant supported detectable telomere resolution at both 12.5 and 6.25 nM TelA concentrations in metal-free conditions. Addition of MgCl_2_ was still stimulatory (3.8- and 7.65-fold stimulation relative to metal-free conditions) implying that a substantial degree of autoinhibition also still exists in this mutant. Finally, the D202R/E337K double mutant (DREK) was assayed and was found to promote rapid telomere resolution at both 12.5 and 6.25 nM TelA concentrations in metal-free conditions. Addition of MgCl_2_ was mildly stimulatory (1.6- and 4.2-fold stimulation relative to metal-free conditions) implying there had been an additive effect of the two mutations in lowering the divalent metal ion dependence of the telomere resolution reaction (**Fig 6B**).

### The D202 and D398 residues in TelA help to suppress hp telomere fusion

**Fig 7:**
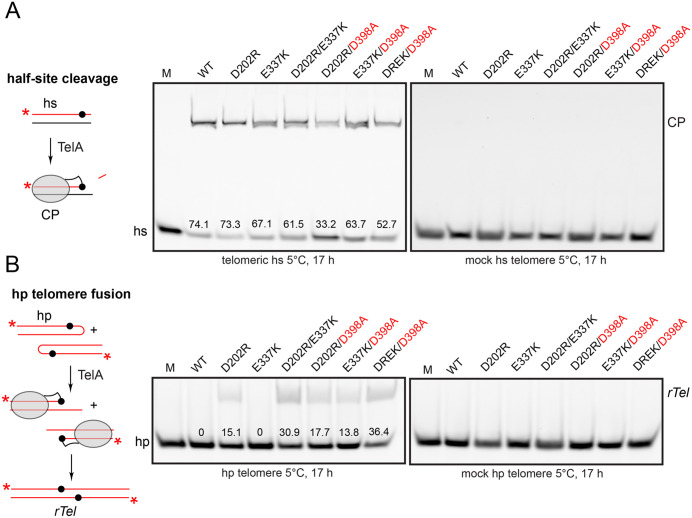
Testing the ability of the TelA mutants to promote reaction reversal. **A)** Half-site cleavage assay of the TelA mutants. Left: shown is a graphic depicting the cleavage assay. When a telomeric half-site is employed TelA cleavage results in a covalent linkage of TelA to the DNA that can be visualized as a bandshift in SDS-containing polyacrylamide (SDS-PAGE) gels. The asterisk indicates a 5’-FITC based fluorophore for visualization of the DNA in the gels. Right: 8% PAGE 1X TAE/0.1% SDS gels of reactions containing 150 nM TelA incubated at 5^o^C for 17 **h.** The numbers above the substrate bands in each lane indicate the percentage of cleavage product obtained in the reaction. hs denotes the half-site; CP denotes the cleavage product. **B)** hairpin (hp) telomere fusion assay. Left: shown is a graphic of the hp telomere fusion assay. Fusion of a pair of hp’s leads to formation of a replicated telomere junction (*rTel*). This is a reversal of the telomere resolution reaction. Right: 8% PAGE 1X TAE/0.1% SDS gels of reactions containing 150 nM TelA incubated at 5^o^C for 17 h**.** The numbers just above the substrate bands in each lane indicate the percentage of reaction reversal obtained in the reaction. hp denotes the hairpin telomere substrate; *rTel* denotes the replicated telomere product of fusion of two hp telomeres. A version of the graphics originally appeared in our previous paper, held under a creative commons license (ref [13]).

Autoinhibition of TelA confers divalent metal ion responsiveness to the telomere resolution reaction and suppresses the cryptic and potentially competing reactions of recombination (the main subject of this study) and hp telomere fusion, a reversal of the telomere resolution reaction [13]. We assessed whether the activating mutations D202R, E337K and their combination with the D398A mutation promoted the latent competing reaction of hp telomere fusion. **Fig 7A** documents reactions with suicide telomeric half-sites that tests the ability of TelA to promote DNA cleavage. Wild type TelA and all the mutants were positive in this assay. The sequence specificity was confirmed by the negative results obtained with a mock telomeric half-site substrate. The ability to fuse hp telomeres of identical sequence was tested and all the TelA variants were positive for this assay excepting wild type TelA and the activated E337K mutant. The sequence specificity was confirmed by the negative results obtained with a mock telomeric hp substrate (**Fig 7B**). The results from this assay and other activities of the mutants indicate that the D202 and D398 residues of the wild type enzyme participate in suppressing the competing reactions of hp telomere fusion and recombination between *rTel*s. The activating effect of the E337K mutation had previously been attributed, primarily, to increased affinity for substrate DNA rather than via interruption of autoinhibitory interactions ([11]).

## Discussion

Previous results revealed that the agrobacterial telomere resolvase, TelA, could be mutated into a Cre-like recombinase by the combination of deletion of autoinhibitory domains and point mutations [12]. Two mutants, in particular, were revealed as complete ‘switch’ mutants that could no longer promote telomere resolution but promoted an atypical Cre-like recombination between *rTel* junctions; (ΔC3; D202R/E337K/D398A) and (ΔN; D202R/E337K/D398A). In the present study we show that only the combination of the D202R and D398A mutations is necessary to produce a TelA mutant that is unable to promote telomere resolution but instead has become activated as an atypical Cre-like recombinase. What follows is a review of what is known about the role of these two aspartic acids in TelA.

**D202:** This aspartic acid residue was highlighted in a crystallographic study of an intermediate in the TelA-mediated process of refolding the cleaved strands into a hairpin conformation. In such a refolding intermediate the D202 residue makes water-mediated contacts with the A6/A6’ nucleotides that are captured in an enzyme-stabilized extrahelical conformation [8]. We were interested in exploring the phenotype of mutants in this residue. We discovered that a D202A mutant was mildly activated as a telomere resolvase and that its activity was less dependent upon the presence of divalent metal ions than wild type TelA [13]. Subsequent analysis revealed that TelA is subject to intrinsic negative regulation of its activity through autoinhibition via its dispensable N-terminal domain and via the latter part of the C-terminal helix [11,13]. Knowledge of these regulatory auto-interactions allowed the design of hyperactive telomere resolvases [11]. An AlphaFold2 modeling exercise seemed to reveal a possible role for D202 in establishing a regulatory interaction with one of 3 positively charged residues that comprise the last 4 amino acids of the C-terminal helix. As part of the effort to test this putative electrostatic interaction we characterized charge reversal mutants that, ultimately, did not support the specific predictions made by the AlphaFold2 model. Nonetheless, the resulting D202R mutant proved to be a useful mutant showing even greater hyperactivity than the D202A mutant and a tendency to be additive with other activating mutations [11]. S2B Fig in S1 File shows a PyMol model of the D202R mutation adding polar contacts with the DNA backbone between the scissile phosphates possibly explaining its hyperactivity in telomere resolution. In addition to possibly stabilizing the product complex, thus favouring the forward trajectory of the telomere resolution reaction, the charge reversal may allow a more direct role in changing strand trajectories after DNA cleavage to promote more rapid strand exchanges to produce hp telomeres or recombinants, depending on the genetic context of the rest of TelA. The D202 residue is absent from ResT. In an alignment of TelA with ResT the D202 residue is present in TelA in a region in the ‌‌alignment that has a gap of several amino acids in ResT [23].

**D398:** In a general mutational survey of highly conserved residues in telomere resolvases an early study of the borrelial telomere resolvase, ResT, revealed that the homologue of this residue (D328) to be part of a network of ResT residues involved in stabilizing an underwound pre-cleavage intermediate in telomere resolution that helps propel the reaction forwards toward hp telomere product formation in preference to DNA cleavage reversing back to substrate [16]. The borrelial D328A mutant was found to be cleavage competent but to be locked into abortive cycles of DNA strand cleavage and rejoining that regenerated the substrate DNA. This and other reported phenotypes led to the proposal that this aspartic acid residue is implicated in ejecting the cleaved strands away from the scissile phosphates to prevent immediate strand resealing to regenerate substrate [16]. Mutation of the equivalent residue in TelA to produce the TelA (D398A) mutant also produced a hypoactive telomere resolvase. When tested on negatively supercoiled plasmid substrates, the D398A mutant seemed to have become a recombinase [14,24]. Combination of this mutation with mutations that hyperactivate telomere resolution produced the first recombinase only ‘switch’ mutants of a telomere resolvase [12]. S2C Fig in S1 File shows a PyMol model of the polar interactions with the substrate DNA made by the D398 residue in a TelA product complex structure [8]. In this context the D398 residue makes water-mediated contacts with the DNA backbone very close to the scissile phosphate. In the pre-cleavage intermediate of ResT and TelA we speculate that a more direct clash of negative charges between D398 and the backbone near the cleavage site promotes strand ejection towards hp products. Loss of this strand ejection energy through charge neutralization in (aspartic acid to alanine mutation) may promote, instead, a more modest strand exchange to produce recombinants in the context where the *rTel* junctions are present in negatively supercoiled DNAs and a synaptic complex of a pair of *rTel*s. While D398 in the catalytic domain is highly conserved in the characterized telomere resolvases it is not conserved in the broad family of tyrosine recombinases [23,25]. The specific sequence conservation with this broad family is reserved to key residues in the active site. Even in this case there is some diversity of permissible residues at some active site residues [2].

The kind of molecular details discussed above would be expected to be insufficient, on their own, to explain the switch phenotype of the D202R/D398A mutant. We expect that there must also be an accompanying change in the higher order arrangement of TelA bound to the *rTel* junctions that comes with the switch mutant. Telomere resolution requires the assembly of a dimer on a single *rTel* junction while recombination should require assembly of dimers on two *rTel* junctions followed by synapsis of the pair of TelA-bound *rTel*s. A detailed investigation of the ability/tendency of wild type TelA *vs*. the D398A and D202R/D398A mutants to oligomerize in the presence or absence of substrate DNA and to effect the synapsis of bound *rTel*s together should help unravel the molecular basis of the ‘switch’ phenotype.

## Supporting information

S1 Fig Raw images. (PDF)

S1 File Supporting information. (PDF)
