# Supplementary material for: Minimal mutational requirements for conversion of a telomere resolvase into a Cre-like site-specific recombinase
Source: PLoS One. 2026 May 29;21(5):e0350834. doi: 10.1371/journal.pone.0350834 (PMC13221069; doi:10.1371/journal.pone.0350834)
Supplement: S1 Fig — (PDF) [file pone.0350834.s001.pdf]

S1 raw images

Fig 2 raw images

A

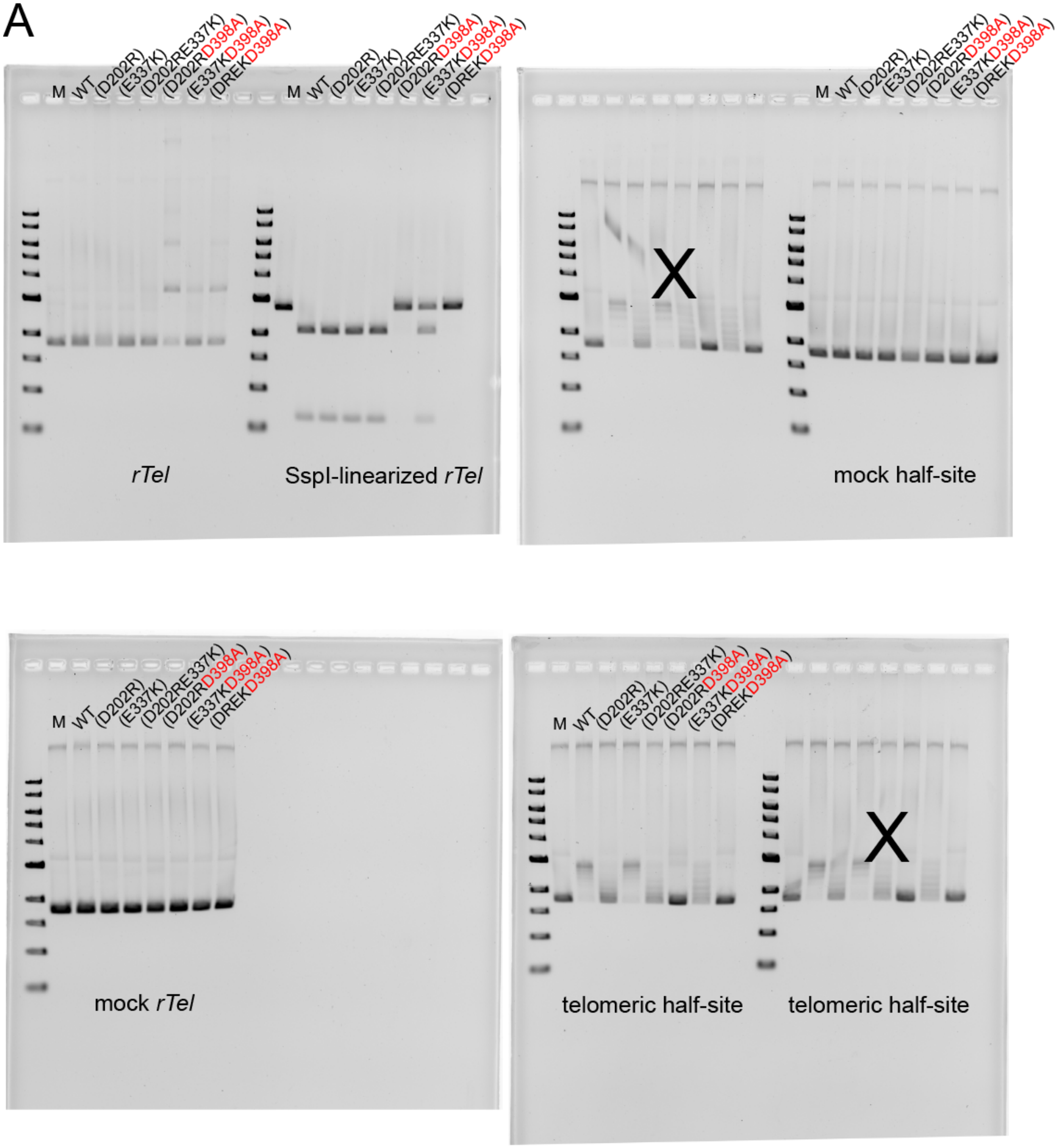

Uncropped gels for Fig 2. Where they appear X’s mark trials not used in this study.

Fig 3BC raw images

A

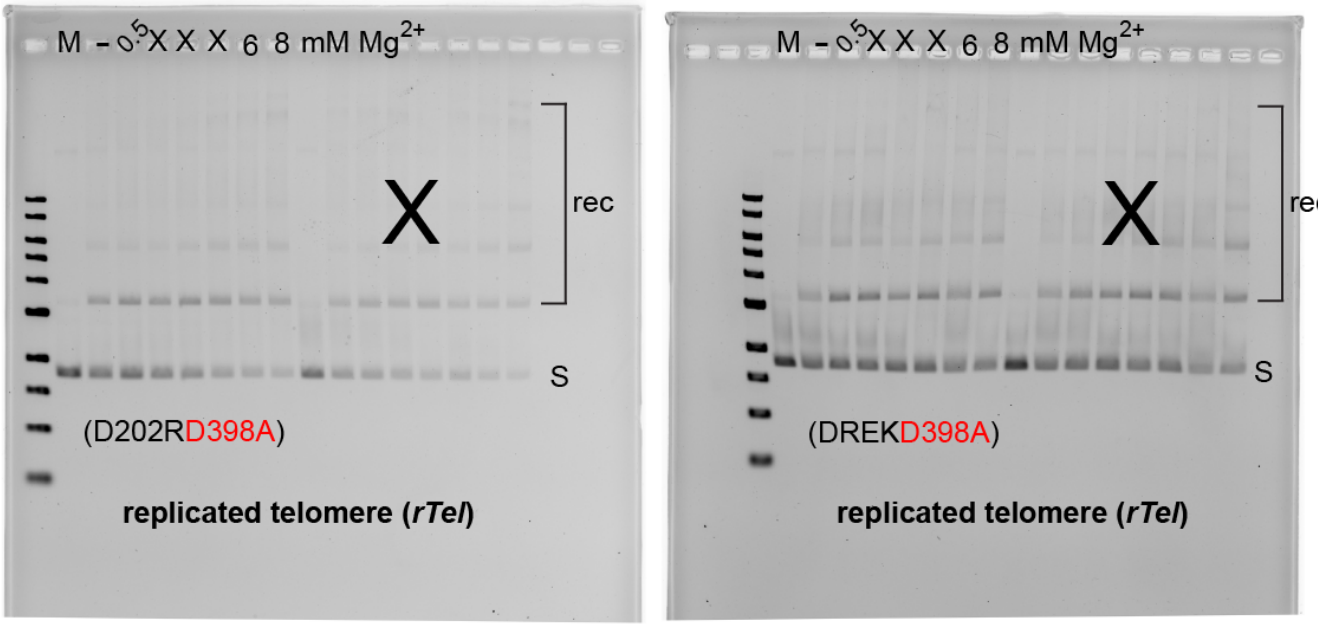

B

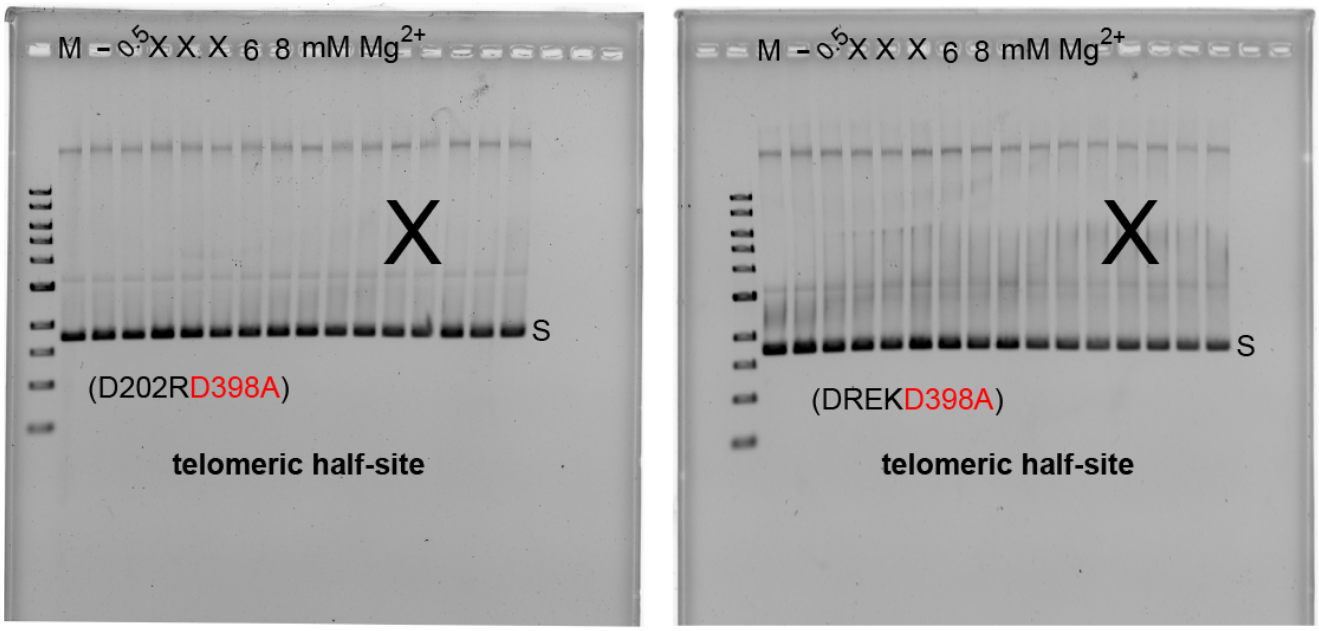

Fig 3D raw images

C

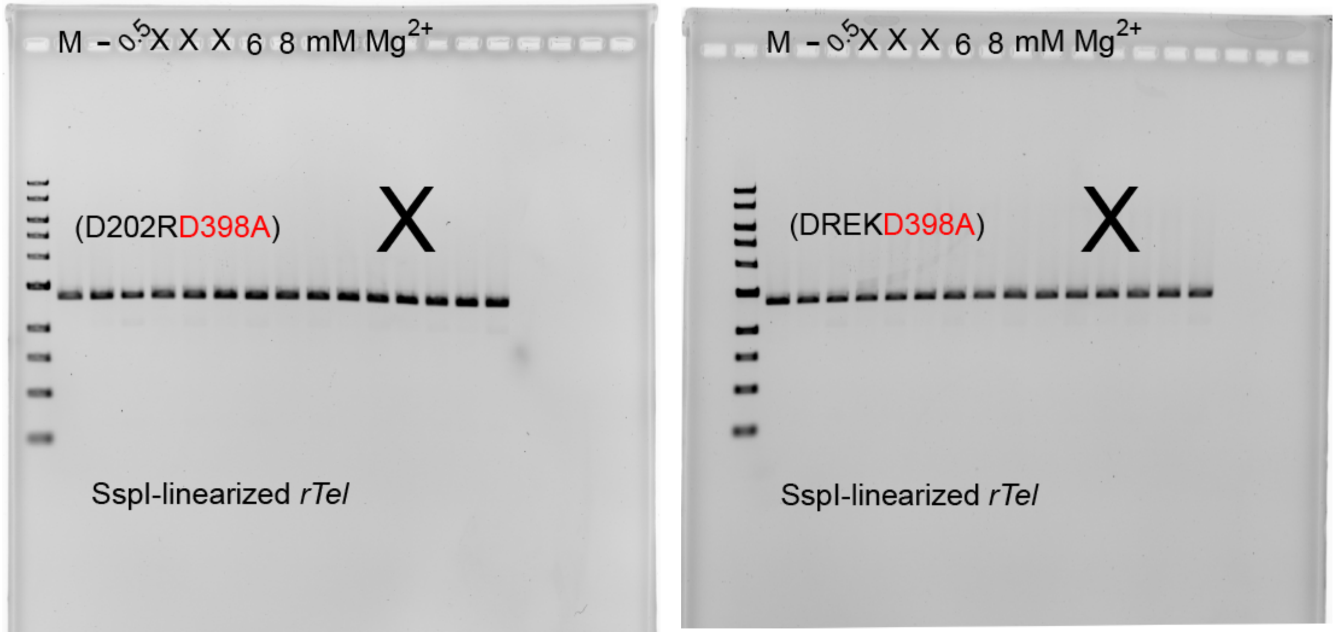

Uncropped gels for Fig 3. Where they appear X's mark divalent metal ion concentrations not reported in this study. The large X's represent panels with titrations of $CaCl_2$ not reported in this study.

Fig 4 raw images

C

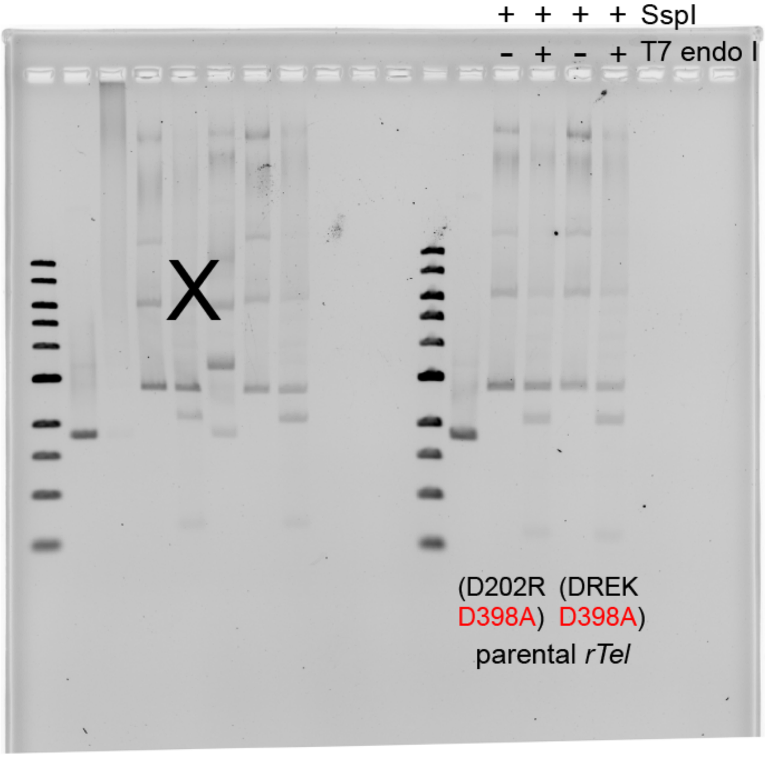

D

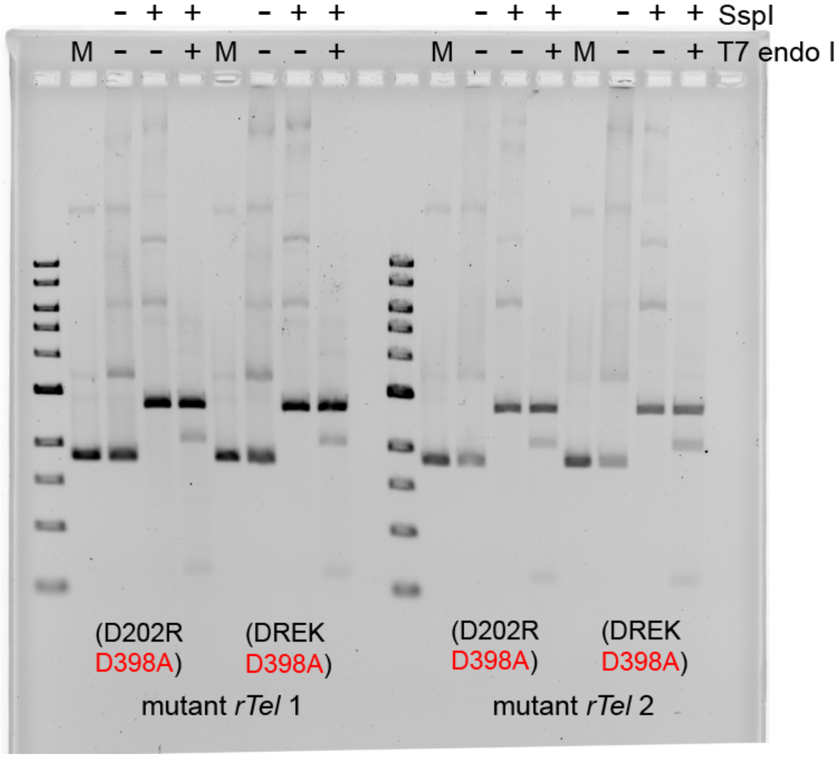

Uncropped gels used in Fig 4. The large X represents a replicated panel not used in this study.

Fig 5 raw images

B

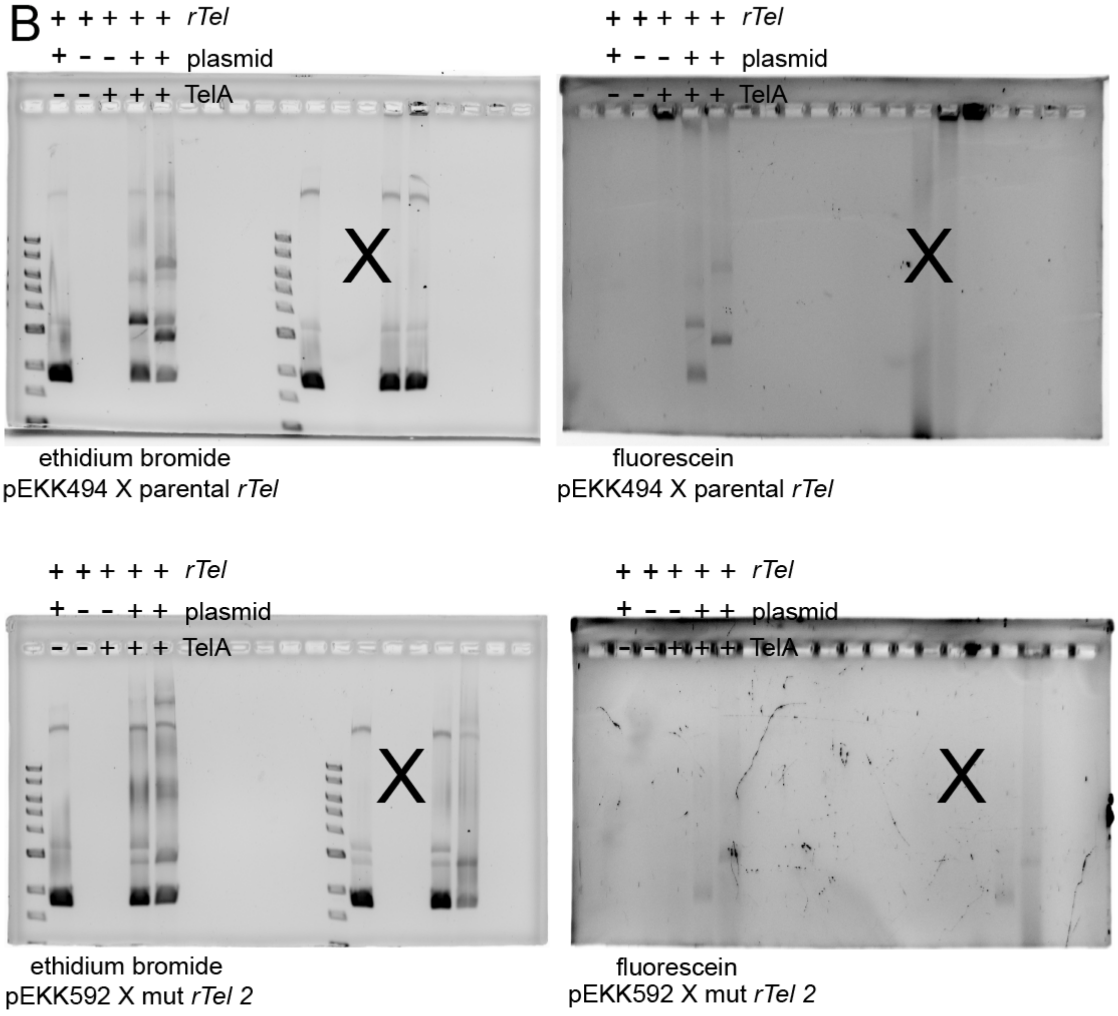

C

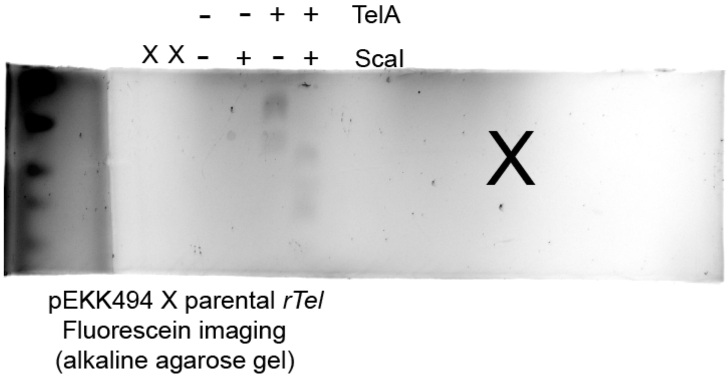

Uncropped gels used in Fig 5. The large X's represent unsuccessful panels with pEKK592 not reported in this study. The small X's in C represent empty lanes between the 1 knt ladder stained in ethidium bromide while the rest of the gel was imaged only in the fluorescein channel prior to staining.

Fig 7 raw images

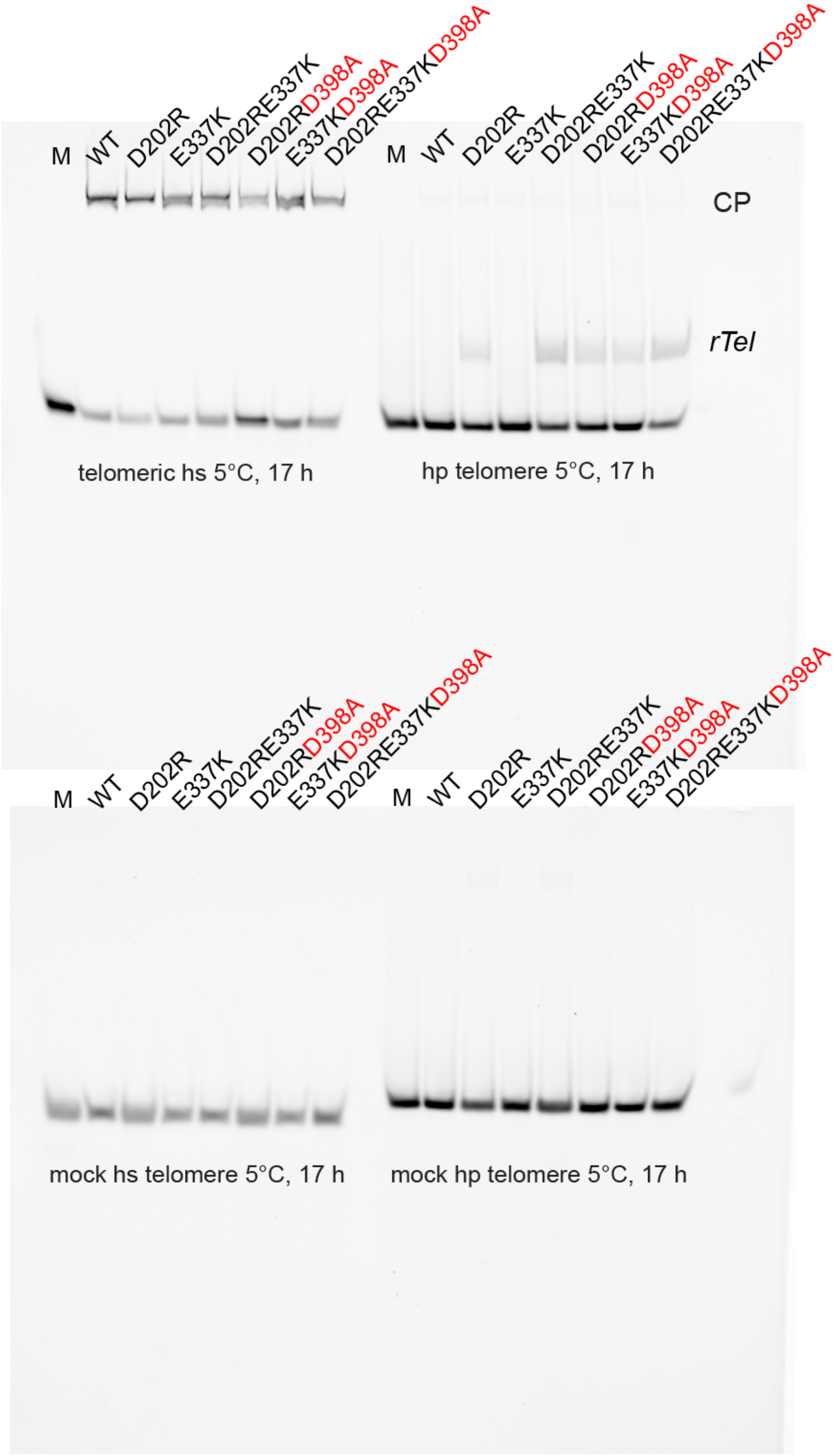

Uncropped gels used for Fig 7.

S3 Fig raw images

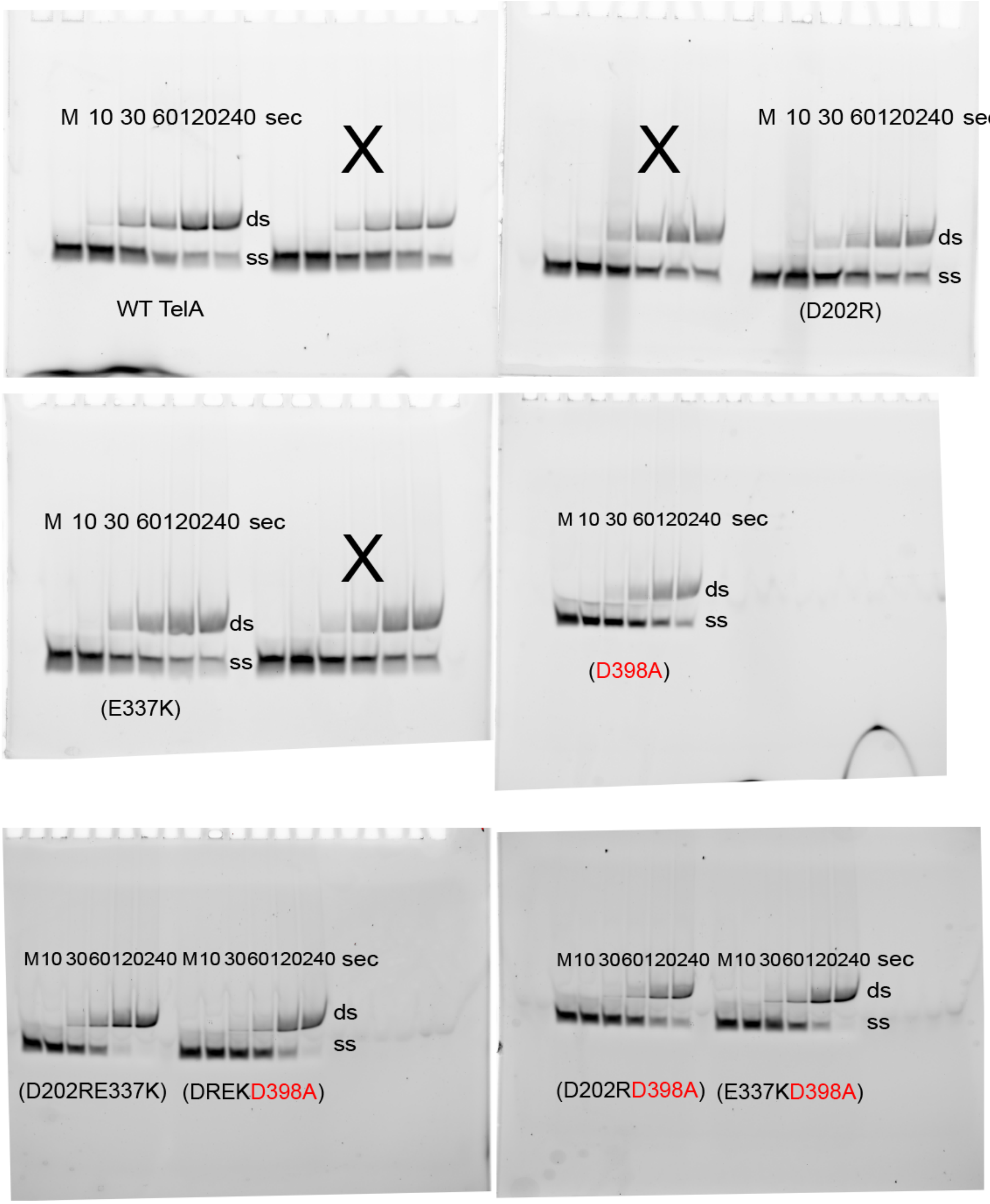

Uncropped gels used for S3 Fig. The large X's represent panels of mutants not used in this study.

S4 Fig raw images

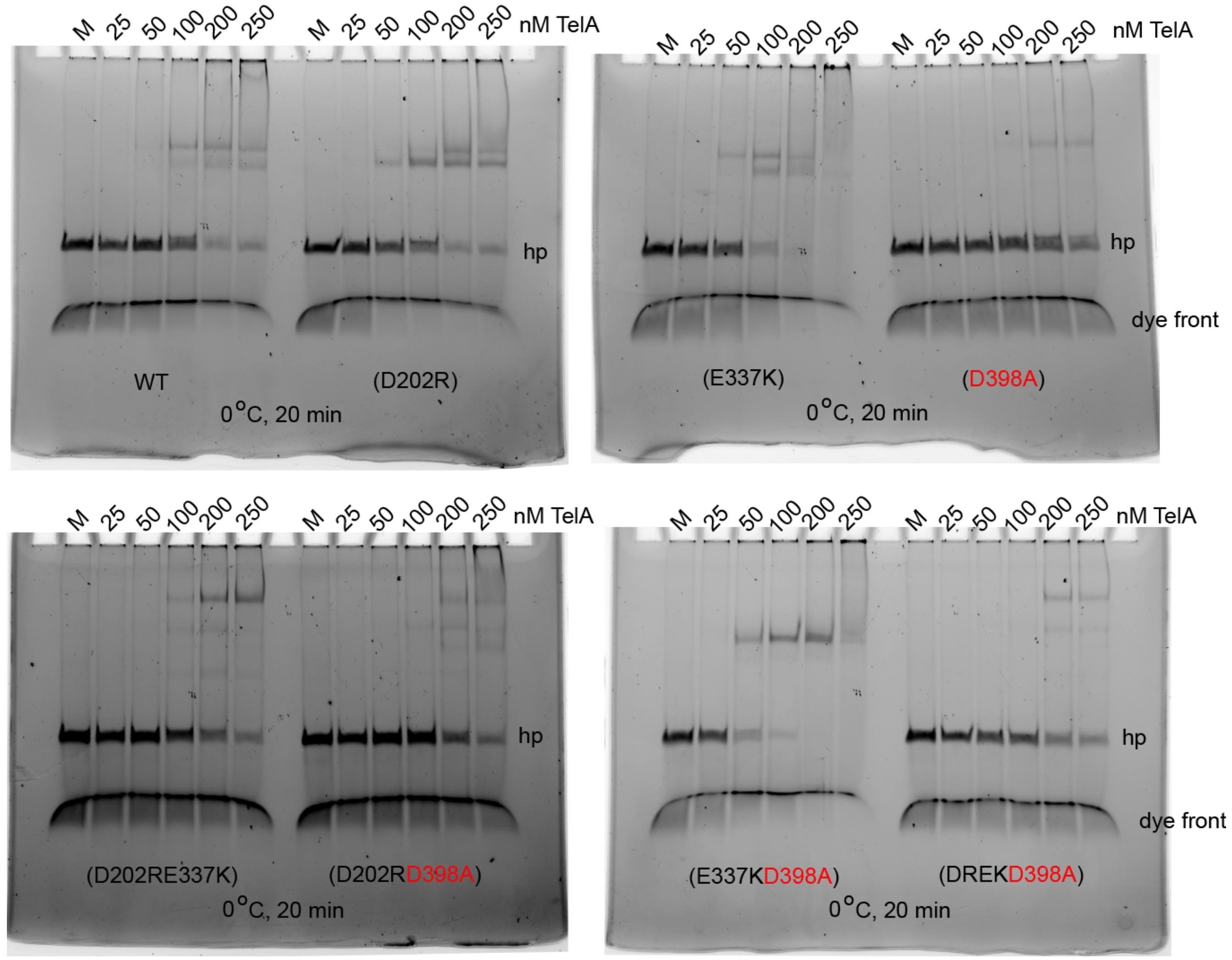

Uncropped gels used for S4 Fig. The bromophenol blue dye front (which fluoresces in the fluorescein channel is also noted.

S5A Fig raw images

A

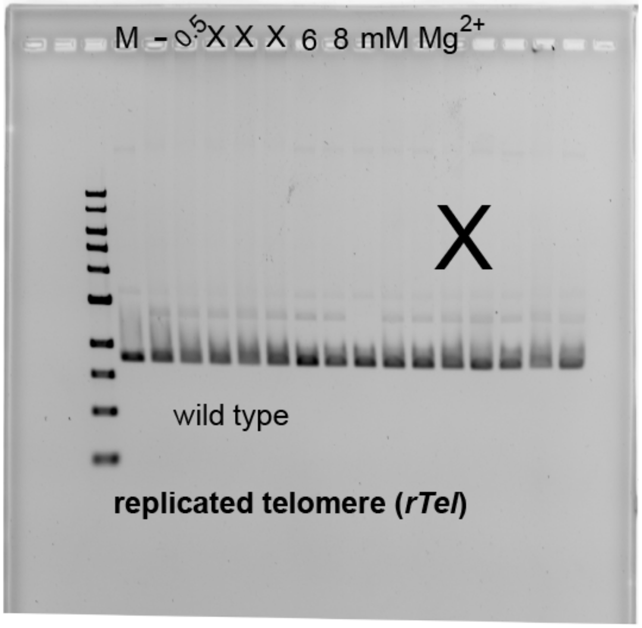

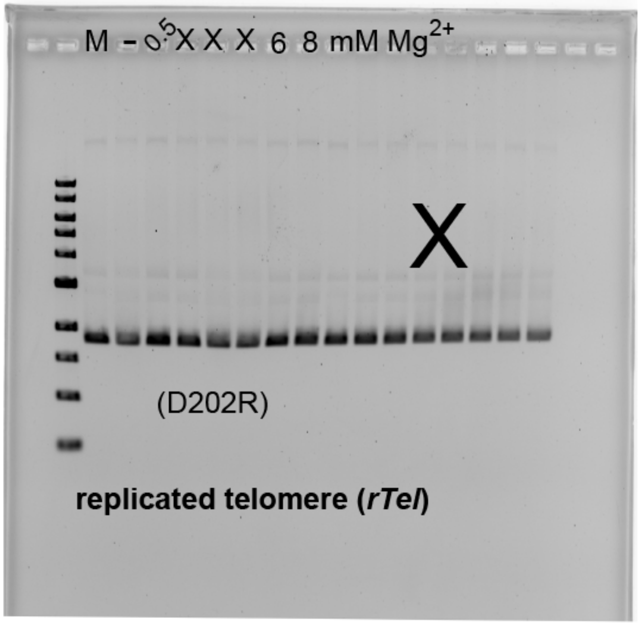

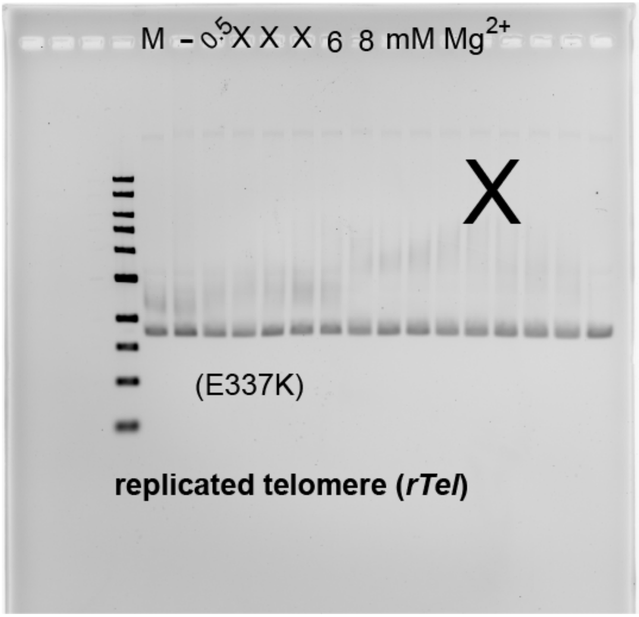

Uncropped gels used in S5A Fig. Where they appear X's mark divalent metal ion concentrations not reported in this study. The large X's represent panels with titrations of $CaCl_2$ not reported in this study.

S5B Fig raw images

B

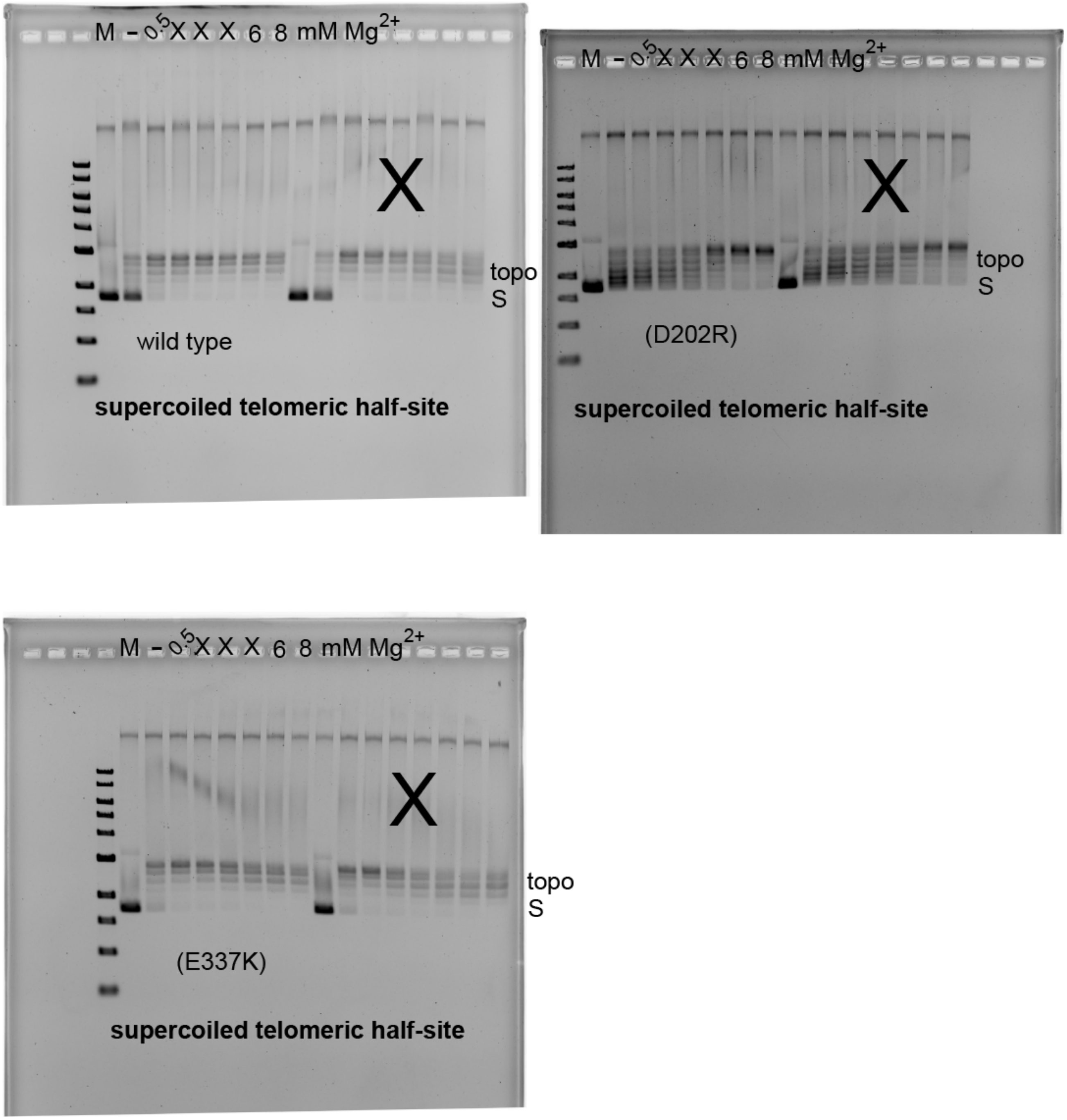

Uncropped gels used in S5B Fig. Where they appear X's mark divalent metal ion concentrations not reported in this study. The large X's represent panels with titrations of $CaCl_2$ not reported in this study.

S5C Fig raw images

C

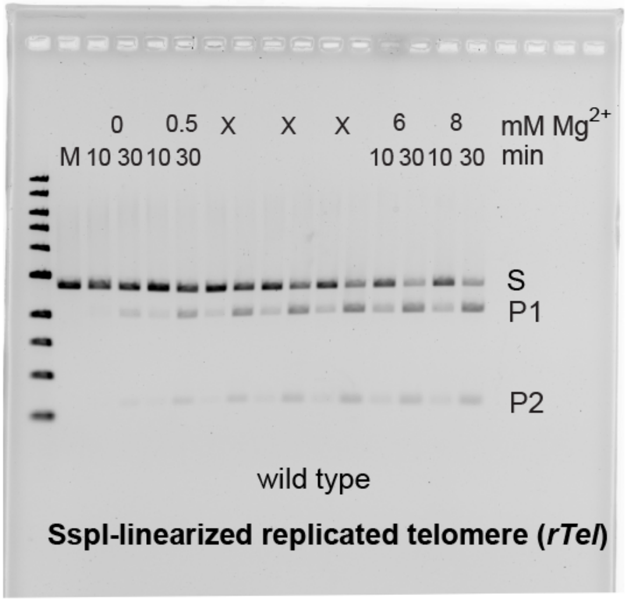

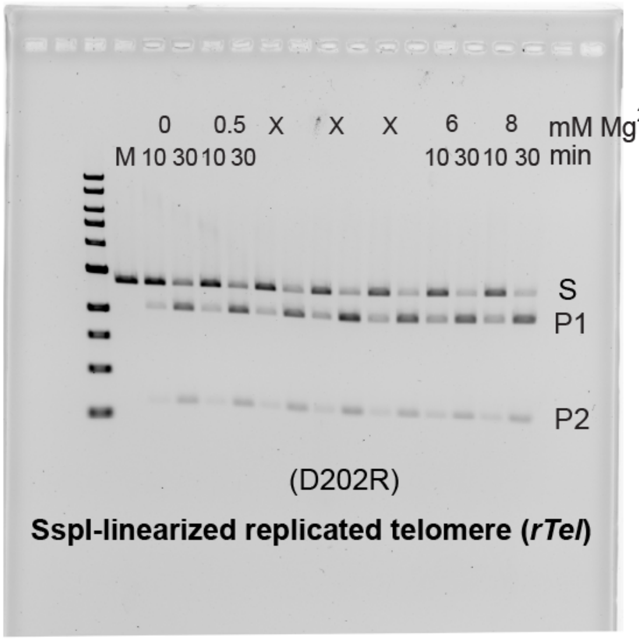

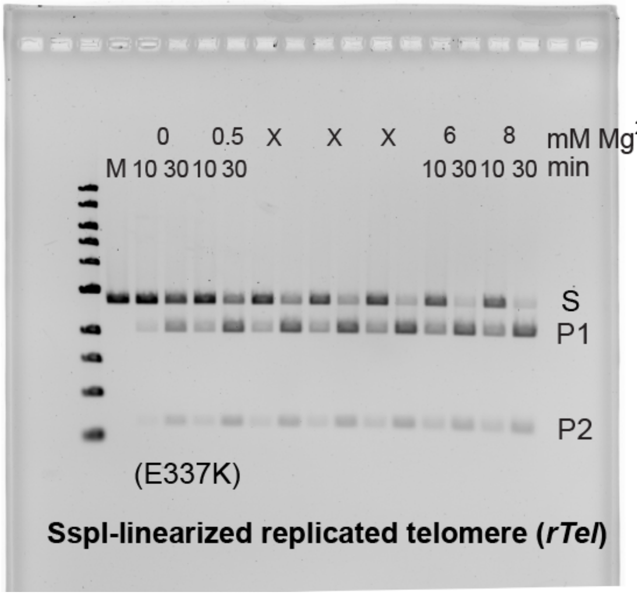

Uncropped gels used in S5C Fig. Where they appear X's mark divalent metal ion concentrations not reported in this study.

S6AB Fig raw images

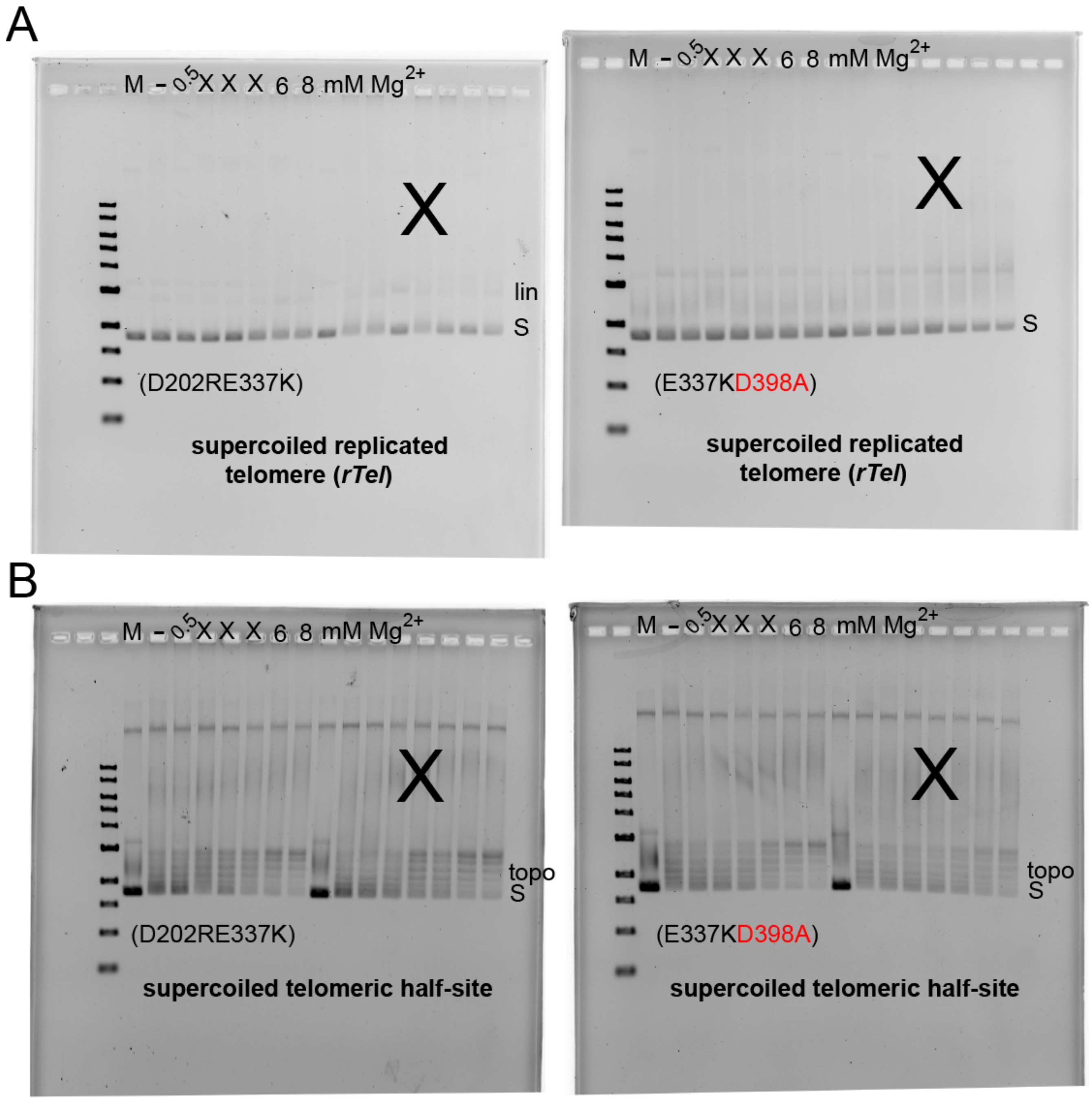

Uncropped gels used in S6AB Fig. Where they appear X's mark divalent metal ion concentrations not reported in this study. The large X's represent panels with titrations of $CaCl_2$ not reported in this study.

S6C Fig raw images

C

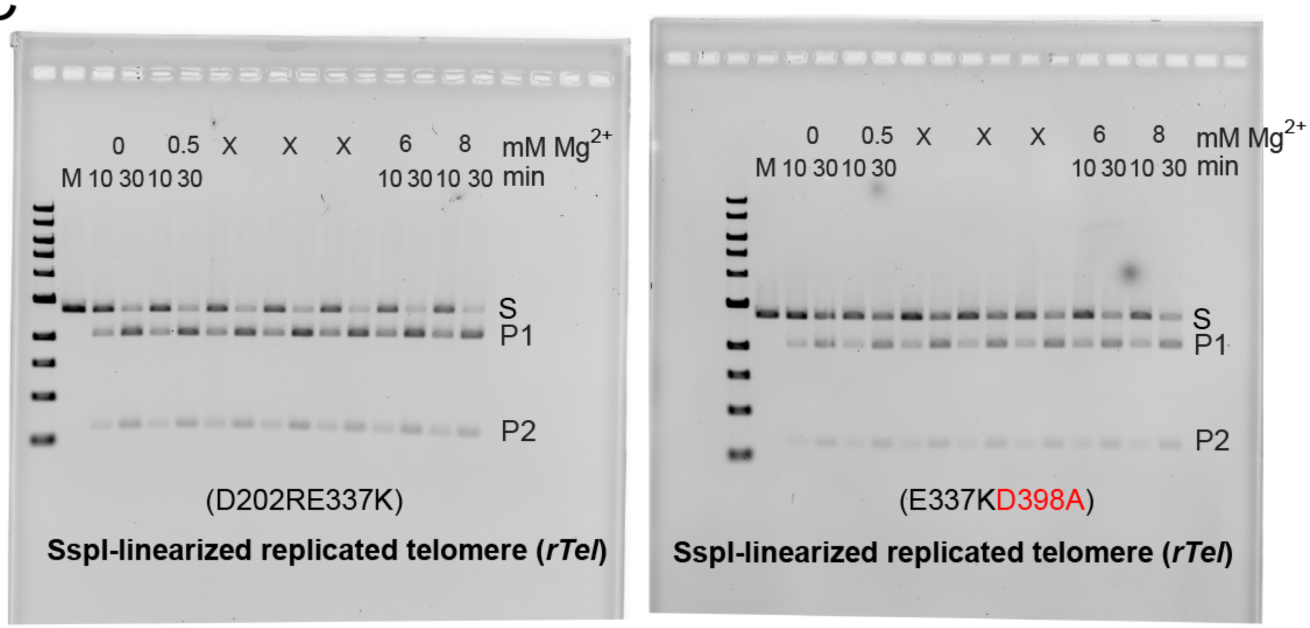

Uncropped gels used in S6C Fig. Where they appear X's mark divalent metal ion concentrations not reported in this study.
