# Supplementary material for: Minimal mutational requirements for conversion of a telomere resolvase into a Cre-like site-specific recombinase
Source: PLoS One. 2026 May 29;21(5):e0350834. doi: 10.1371/journal.pone.0350834 (PMC13221069; doi:10.1371/journal.pone.0350834)
Supplement: S1 File — (PDF) [file pone.0350834.s002.pdf]

Supporting information

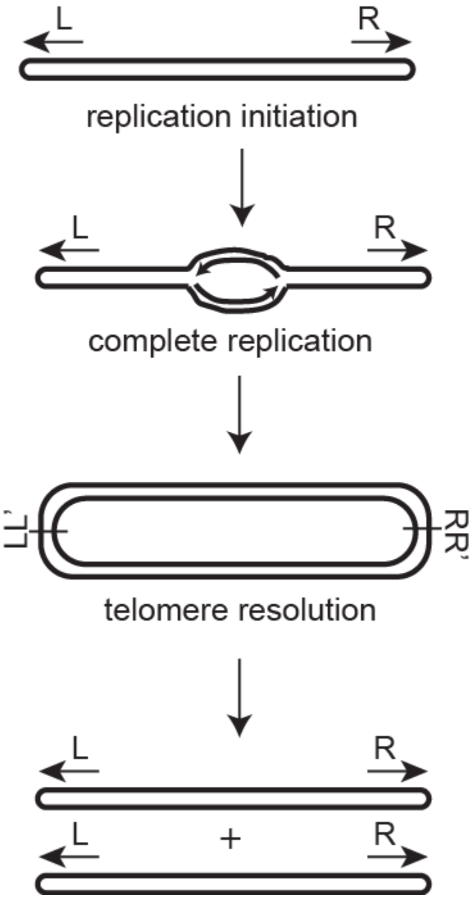

S1 Fig. Replication pathway of linear DNAs terminated by hairpin (hp) telomeres. Initiation of replication occurs at an internal *oriC* sending two replication forks out from the origin towards the hairpin (hp) telomeres. Replication around the hp turnarounds completes replication to produce a circular inverted repeat replicon dimer that is then resolved into two linear DNA's terminated by hp telomeres by a DNA cleavage and rejoining reaction referred to as telomere resolution.

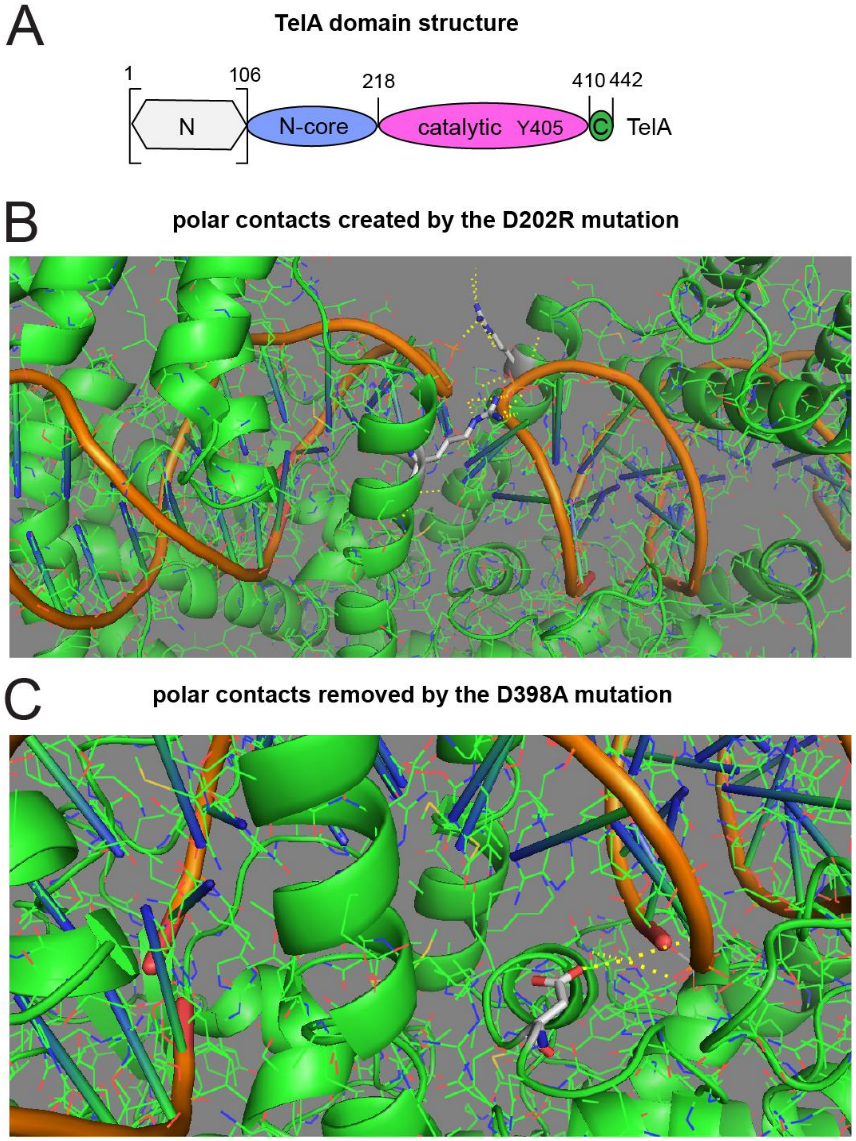

S2 Fig. Molecular modeling of the activating/switching TelA mutations.
A) Domain structure of TelA is shown. The autoinhibitory N-terminal domain is shown in shaded white, the N-core domain in blue, the catalytic domain is coded as pink and the C-terminal helix in green. The latter part of the C-terminal helix also contributes to autoinhibition. A version of the graphic originally appeared in our previous paper, held under a creative commons license (ref [1]).
B) Molecular modeling of the protein-DNA contacts gained in the D202R mutation.
The model was built with PyMol using pdb accession# 4e0g.
C) Molecular modeling of the protein-DNA contacts lost in the D398A mutation. The model is derived from a co-crystal structure of TelA with hairpin telomere DNA. The scissile phosphate is shaded in red, the red crosses represent water molecules and the dashed lines the polar contacts. The model was built with PyMol using pdb accession# 4e0g.

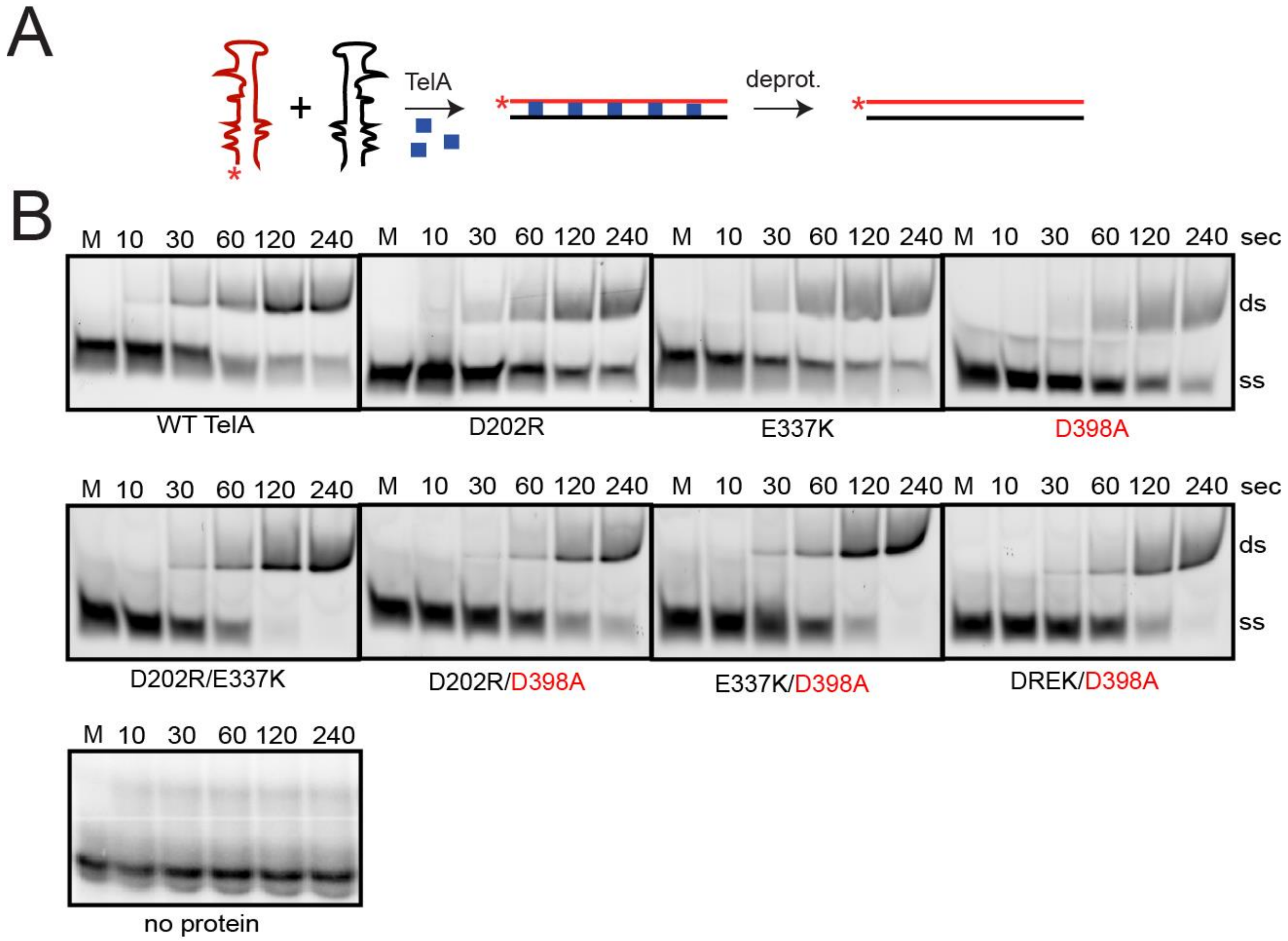

S3 Fig. Single-stranded DNA annealing assays of the TelA variants.
A) Graphic representing the ssDNA annealing assay using synthetic DNA oligonucleotides comprising the HIV_{TAR} element. This sequence element possesses significant secondary structure that strongly suppresses spontaneous annealing. The 5'-fluorescein endlabeled reporter strand is shown in red and the complementary, unlabeled strand is shown in black. Formation of the deproteinated double-stranded product was visualized by polyacrylamide gel electrophoresis. A version of the graphic originally appeared in our previous paper, held under a creative commons license (ref [2]).
B) 8% PAGE 1X TAE/0.1% SDS gel panels of annealing timecourse reactions with the indicated TelA variants; a no protein annealing reaction is shown in the final panel.

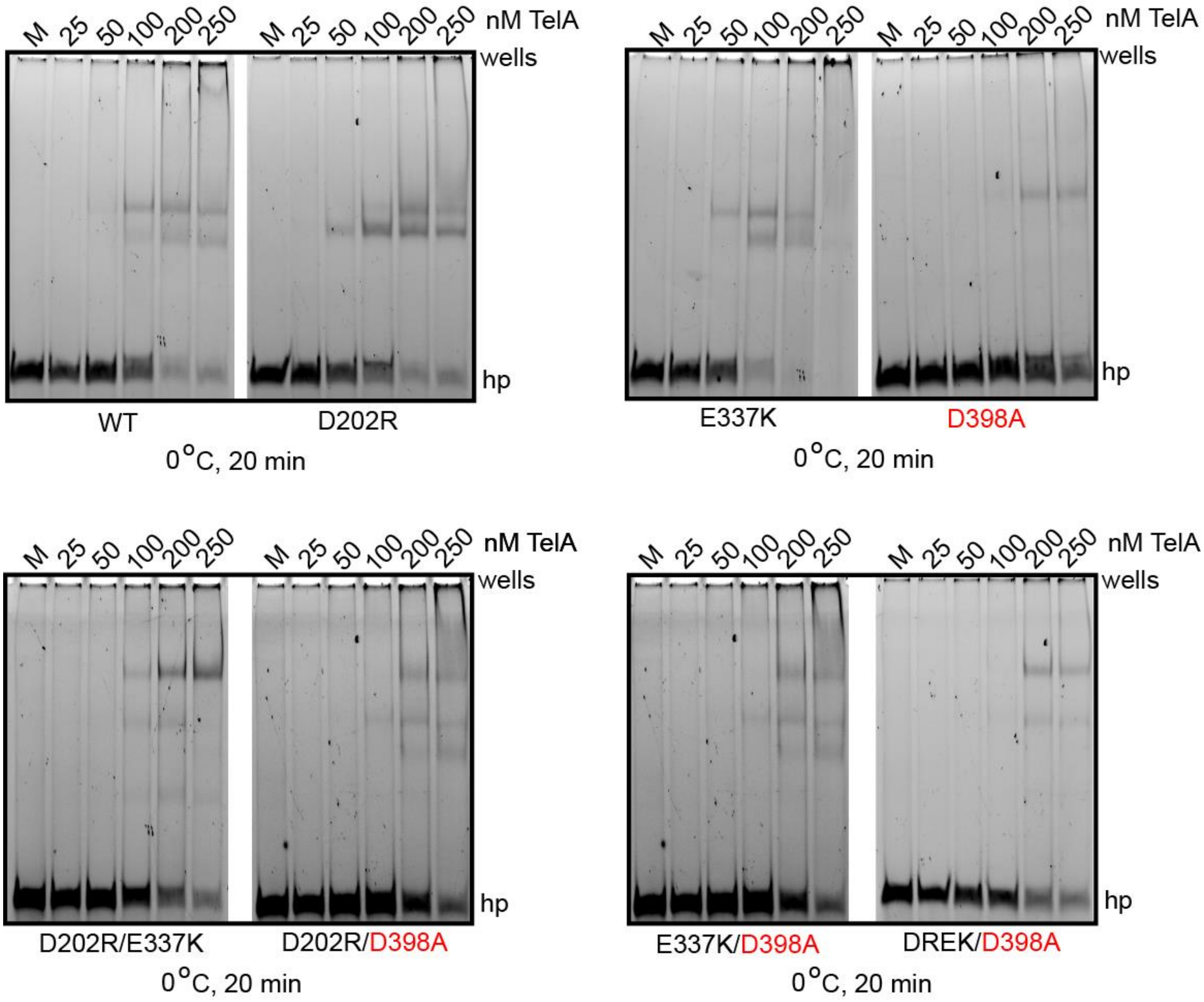

S4 Fig. Electrophoretic mobility shift assays (EMSA) of the TelA variants.
6% PAGE 0.5X TBE gel panels of wild type TelA the indicated TelA mutants with a 5'-fluorescein endlabeled hp telomere. Binding to the hairpin product was assessed instead of the *rTel* substrate to avoid the resolution of the *rTel* promoted by the activated mutants.

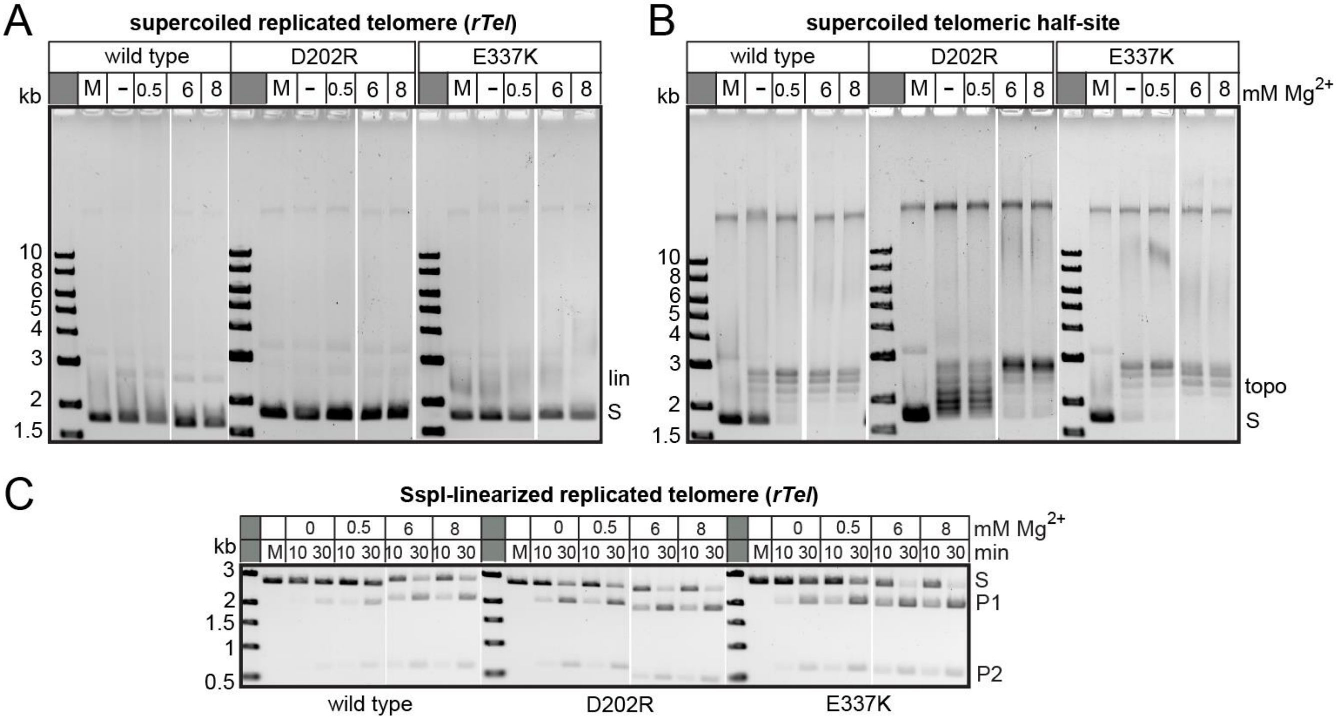

S5 Fig. Testing the divalent metal ion responsiveness of the D202R and E337K mutants relative to wild type TelA.

A) 0.8% agarose 1X TAE gel panels of divalent metal ion titrations of wild type TelA, the D202R and the E337K mutants reacted with negatively supercoiled plasmid substrate (pEKK494) incubated at 30°C for 30 min in a buffer containing the indicated concentrations of $MgCl_2$. The 0, or no Mg^{2+} condition contains 1 mM EDTA instead. S denotes substrate; lin denotes the position that the linear product of telomere resolution; topos, denotes the ladder of topoisomers.

B) 0.8% agarose 1X TAE gel panel of divalent metal ion titrations of wild type TelA, the D202R and the E337K mutants reacted with negatively supercoiled telomeric half-site plasmid (pEKK588) incubated at 30°C for 30 min in a buffer containing the indicated concentrations of $MgCl_2$.

C) 0.8% agarose 1X TAE gel panels of divalent metal ion titrations of wild type TelA, the D202R and the E337K mutants reacted with SspI-linearized *rTel* plasmid (pEKK494) incubated at 30°C for 30 min in a buffer containing the indicated concentrations of $MgCl_2$. S denotes substrate; P1 & P2 denote the migration position of the expected products of telomere resolution.

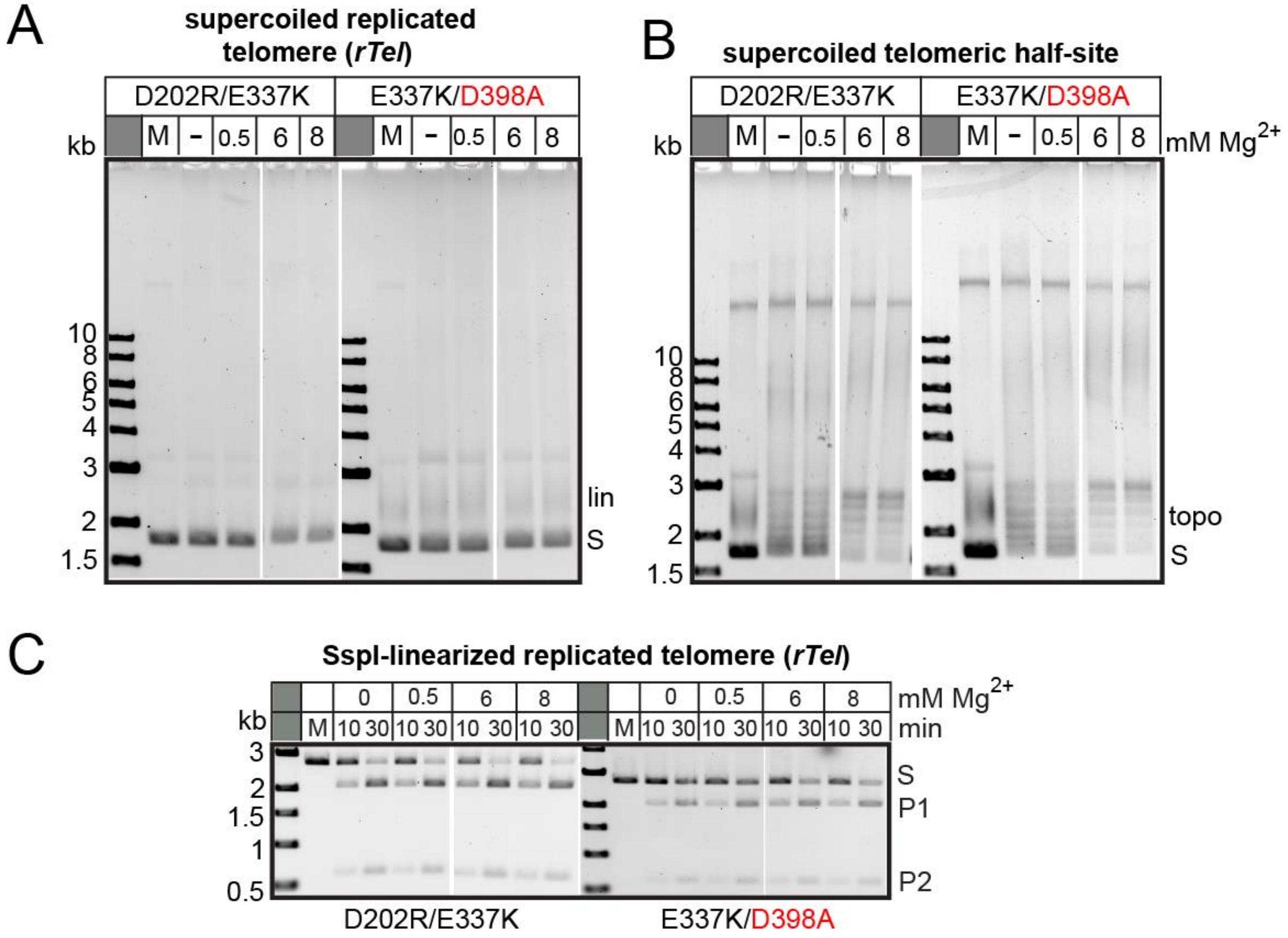

S6 Fig. Testing the divalent metal ion responsiveness of the D202R/E337K and E337K/D398A mutants.
A) 0.8% agarose 1X TAE gel panels of divalent metal ion titrations of the D202RE337K and E337KD398A mutants reacted with negatively supercoiled plasmid substrate (pEKK494) incubated at 30°C for 30 min in a buffer containing the indicated concentrations of $MgCl_2$. The 0, or no Mg^{2+} condition contains 1 mM EDTA instead. S denotes substrate; lin denotes the position that the linear product of telomere resolution; topos, denotes the ladder of topoisomers.
B) 0.8% agarose 1X TAE gel panel of divalent metal ion titrations of the D202RE337K and E337KD398A mutants reacted with negatively supercoiled telomeric half-site plasmid (pEKK588) incubated at 30°C for 30 min in a buffer containing the indicated concentrations of $MgCl_2$.
C) 0.8% agarose 1X TAE gel panels of divalent metal ion titrations of the D202RE337K and E337KD398A mutants reacted with SspI-linearized *rTel* plasmid (pEKK494) incubated at 30°C for 30 min in a buffer containing the indicated concentrations of $MgCl_2$. S denotes substrate; P1 & P2 denote the migration position of the expected products of telomere resolution.

S1 Table. Oligonucleotides used in this study.

Oligo name	Oligo sequence	Use
OGCB878	5’-CTGGGCCACAACAATAATGCGTTAGAAACAAGTCTTTCT-3’	Make D398A; ts; with WT and mutant parents
OGCB879	5'-AGAAAGACTTGTTTCTAACGCATTATTGTTGTGGCCCAG-3'	Make D398A; bs; with WT and mutant parents
OKBA19	5'-acagcagacgtgtcttggacgagaagtcgtc-3'	Make E337K; bs; WT parent and D398A parent
OKBA20	5'-gacgacttctcgtccaagacacgtctgctgt-3'	Make E337K; bs; WT parent and D398A parent
OKBA27F	5’F-atcCCTCTAACCATTGCGCGATCGATCATAATAACAATATCA-3’	5’-fluorescein labeled ts half-site
OGCB871	5’-TGATATTGTTATTATGATCGATCGCGCAATGGTTAGAGG-3’	bs half-site
OKBA28F	5’-atcCCTCTAACCATTGCGCGATCGATCATAATAAC AATATCATGATATTGTTATTATGATCGATCGCGCAATGGTTAGAGG-3’	5’-fluorescein labeled hairpin telomere
OKBA29F	5’-atcGGAGATTGGTAACGCGCTAGCATGTATTATTGTTATAGT-3’	5’-fluorescein labeled ts mock half-site
OGCB914	5’-CCTCTAACCATTGCGCGATCGTACATAATAACAATATCA-3’	bs mock half-site
OKBA30F	5’-atcGGAGATTGGTAACGCGCTAGCATGTATTATTGTTATAGTA CTATAACAATAATACTAGCATGCGCGTTACCAATCTCC-3’	5’-fluorescein labeled mock hairpin telomere
OGCB951F	5’F-aaCTCTAACCATTGCGCGATCGATCATAATAACAATATCATGATATTGTTATTGTAATCGATCGCGGATCCCGGGCGTAGCCACGTAGGT-3’	5’-fluorescein labeled ts *rTel*
OGCB952	5’-gaACCTACGTGGCTACGCCCGGGATCCGCGATCGATTACAATAAC AATATCATGATATTGTTATTATGATCGATCGCGCAATGGTTAGAG-3’	bs *rTel*
OKBA45F	5’F-aaGAGATTGGTAACGCGCTAGCTAGTATTATTGTTATAGTACTATAACA ATAACATTAGCTAGCGCCTAGGGCCCGCATCGGTGCATCCA-3’	5’-fluorescein labeled ts mock *rTel*
OKBA46	5’-gaTGGATGCACCGATGCGGGCCCTAGGCGCTAGCTAATGTTATTGTTATA GTACTATAACAATAATACTAGCTAGCGCGTTACCAATCTC-3’	bs mock *rTel*
OGCB951	5’-aaCTCTAACCATTGCGCGATCGATCATAATAACAATATCATGATATTGTT ATTGTAATCGATCGCGGATCCCGGGCGTAGCCACGTAGGT-3’	ts *rTel*
OKBA45	5’-	ts mock *rTel*

	aaGAGATTGGTAACGCGCTAGCTAGTATTATTGTTATAGTACTATAAC AAT AACATTAGCTAGCGCCTAGGGCCCGCATCGGTGCATCCA-3’	
OKBA67	5'-gatcCTCTAACCATTGCGCGATCGATCATAATAACAATACCTTGATAT TGTTA TTGTAATCGATCGCGGATCCCGGCGTAGCCACGTAG-3’	ts asymmetric *rTel* for BamHI-HindIII cloning into pUC19 pEKK592
OKBA68	5’-agctCTACGTGGCTACGCCCGGGATCCGCGATCGATTACAATAACAAT ATCAA GGTATTGTTATTATGATCGATCGCGCAATGGTTAGAG-3’	bs asymmetric *rTel* for BamHI-HindIII cloning into pUC19 pEKK592
OKBA67F	5'F-aaCTCTAACCATTGCGCGATCGATCATAATAACAATACCTTGATATTG TTATTGTAATCGATCGCGGATCCCGGGCGTAGCCACGTAG-3’	ts asymmetric *rTel with* 5’ fluorescein
OKBA68F	5’F-aaCTACGTGGCTACGCCCGGGATCCGCGATCGATTACAATAACAATAT CAAG GTATTGTTATTATGATCGATCGCCAATGGTTAGAG-3’	bs asymmetric *rTel with* 5’ fluorescein
OKBA69	5'-gatcCTCTAACCATTGCGCGATCGATCATAATAACAATACATTGATAT TGTTA TTGTAATCGATCGCGGATCCCGGGCGTAGCCACGTAG-3’	ts asymmetric *rTel* for BamHI-HindIII cloning into pUC19 pEKK592
OKBA70	5’-agctCTACGTGGCTACGCCCGGGATCCGCGATCGATTACAATAACAAT ATCAA TGTATTGTTATTATGATCGATCGCGCAATGGTTAGAG-3’	bs asymmetric *rTel* for BamHI-HindIII cloning into pUC19 pEKK592
OKBA51F	5’-AGGTCTCTCTTGTTAGACCAGGTCGAGCCCGGGAGCTCTCTGGCT AGCAAGGAACCC-3’	ts with 5’ fluorescein label for ssDNA annealing
OGCB899	5’-GGGTTCCTTGCTAGCCAGAGAGCTCCCGGGCTCGACCTGGTCTAA CAAGAGAGACC-3’	bs unlabeled strand for ssDNA annealing

S2 Table. Induction/expression conditions for TelA mutants.

Strain #	Mutant	Induction/expression conditions
EKK601	D202RD398A	0.25 mM IPTG induction at 24°C O/N (2L)
EKK602	E337KD398A	0.125 mM IPTG induction at 24°C O/N (1L)
EKK603	D202RE337K	0.125 mM IPTG induction at 24°C O/N (2L)
EKK604	D202RE337KD398A	0.125 mM IPTG induction at 24°C for O/N (2L)

Supplemental Material and Methods

Single-stranded DNA annealing reactions

The annealing assays were performed in a buffer containing 25 mM HEPES (pH 7.6), 1 mM DTT, 2 mM $CaCl_2$, 100 μg/mL BSA, 50 mM potassium glutamate and 15 nM of the 5' fluorescein-endlabeled reporter oligonucleotide (OKBA51F; **S1 Table**). To prevent spontaneous annealing the reactions were assembled on ice prior to addition of 15 nM the complementary oligonucleotide (OGCB899) and 154 nM TelA. Following the addition of TelA the reactions were incubated at 30°C. 18 μL aliquots were taken from the annealing reactions at the indicated timepoints. To stop further annealing, these aliquots were resuspended in an SDS-containing load dye to a 1X final concentration that contained 0.3 μM of the unlabeled reporter oligonucleotide (OKBA51). 1X load dye contains 0.15% SDS, 20 mM EDTA, 3.2% glycerol, and 0.024% bromophenol blue. The oligonucleotides used for the annealing assay are derived from the HIV_{TAR} element [3].

Electrophoretic mobility shift assays (EMSA)

TelA was bandshifted with its hp product in a buffer containing 25 mM HEPES (pH 7.6), 1 mM DTT, 2 mM $CaCl_2$, 100 μg/mL BSA, 50 mM potassium glutamate, 0.8 μg/mL competitor DNA (supercoiled pUC19) and 76 ng/mL heparin sulphate. The indicated concentrations of TelA were incubated at 0°C for 20 min with 2 nM of 5' fluorescein-endlabeled *rTel* assembled from oligonucleotides OGCB951F/952 (see **S1 Table**). After the native load dye was added to a 1X concentration, samples were applieded to 6% PAGE 0.5X Tris-Borate EDTA (TBE) gels followed by electrophoresis at 15V/cm in a 4°C until the dye front was 1 cm from the gel bottom. The gels were visualized for documentation on a BioRad GelDoc system using the UV transilluminator and the fluorescein program. 1X loading dye contains 20 mM EDTA, 3.2% glycerol, and 0.024% bromophenol blue.

Supplemental References

1. McGrath SL, Huang SH, Kobryn K. The N-terminal domain of the Agrobacterium tumefaciens telomere resolvase, TelA, regulates its DNA cleavage and rejoining activities. J Biol Chem. 2022;298(5):101951-64.
2. Huang SH, Abrametz K, McGrath SL, Kobryn K. Design and characterization of hyperactive mutants of the Agrobacterium tumefaciens telomere resolvase, TelA. PLoS One. 2024;19(7):e0307590. Epub 20240725. doi: 10.1371/journal.pone.0307590. PubMed PMID: 39052566; PubMed Central PMCID: PMCPMC11271964.
3. Lapadat-Tapolsky M PC, Borie C and Darlix J-L. Analysis of the nucleic acid annealing activities of nucleocapsid protein from HIV-1. Nucleic Acids Research. 1995;23(1):2434-41.
